# Modelling passive sampling of hydrophilic compounds under time-variable aqueous concentrations

**DOI:** 10.1007/s11356-024-34460-x

**Published:** 2024-08-12

**Authors:** Benjamin Becker, Christian Kochleus, Denise Spira, Julia Bachtin, Fabian König, Stefan Meinecke, Christel Möhlenkamp, Kees Booij

**Affiliations:** 1 Federal Institute of Hydrology (BfG), Am Mainzer Tor 1, 56068 Koblenz, Germany; 2 German Environment Agency (UBA), Schichauweg 58, 12307 Berlin, Germany; 3 PaSOC, Greate Pierwei 25, 8821 LV Kimswerd, The Netherlands

**Keywords:** Passive sampling, Diffusion model, Sampling rate model, Biofouling, Flow effects, Peak events, SDB-RPS, Silicone

## Abstract

Passive sampling is a crucial method for evaluating concentrations of hydrophilic organic compounds in the aquatic environment, but it is insufficiently understood to what extent passive samplers capture the intermittent emissions that frequently occur for this group of compounds. In the present study, silicone sheets and styrene-divinyl benzene-reversed phase sulfonated extraction disks with and without a polyethersulfone membrane were exposed under semi-field conditions in a 31 m^3^ flume at three different flow velocities. Natural processes and spiking/dilution measures caused aqueous concentrations to vary strongly with time. The data were analyzed using two analytical models that account for these time-variable concentrations: a sampling rate model and a diffusion model. The diffusion model generally gave a better fit of the data than the sampling rate model, but the difference in residual errors was quite small (median errors of 19 vs. 25% for silicone and 22 vs. 25% for SDB-RPS samplers). The sampling rate model was therefore adequate enough to evaluate the time-integrative capabilities of the samplers. Sampler performance was best for SDB-RPS samplers with a polyethersulfone membrane, despite the occurrence of lag times for some compounds (0.1 to 0.4 days). Sampling rates for this design also spanned a narrower range (80 to 110 mL/day) than SDB-RPS samplers without a membrane (100 to 660 mL/day). The effect of biofouling was similar for all compounds and was consistent with a biofouling layer thickness of 150 µm.

**Supplementary Information:**

The online version contains supplementary material available at 10.1007/s11356-024-34460-x.

## Introduction

Environmental monitoring of hydrophilic organic compounds in water can be done using grab sampling, continuous water sampling, and passive sampling. Emissions of hydrophilic compounds (for example polar pesticides and pharmaceuticals) often occur as peak concentration events, following rainfall and intermittent emissions of prescription drugs and illicit drugs (Gallé et al. 2020; Harman et al. 2011; Morosini et al. 2017; Ort et al. 2014). Passive sampling can be considered when the cost of high-frequency grab sampling and continuous water sampling is prohibitive. A current weakness of passive sampling of hydrophilic compounds is that the mechanisms of sampler-water exchange kinetics are not well understood, and that reported sampling rates (*R*_s_) and sorption coefficients (*K*_sw_) of these compounds show a large scatter, without having a clear relationship with flow velocity and temperature (Charriau et al. 2016; Harman et al. 2012).

Passive sampler calibration is commonly done by exposing samplers to constant aqueous concentrations (*C*_w_) for time periods of typically 2 weeks, followed by data analysis with the sampling rate model

1 $${N}_{s}={mK}_{sw}{C}_{w}\left(1-\text{exp}\left[-\frac{{R}_{s}t}{m{K}_{sw}}\right]\right)$$

where *N*_s_ is the accumulated amount, *m* is the sampler mass, *K*_sw_ is the sorbent-water sorption coefficient in volume per mass units, and *t* is time. *R*_s_ is obtained from the initial slope of *N*_s_ vs. time, and *K*_sw_ from the plateau at long time scales (Huckins et al. 2006). The time-integrative window (TIW) is a useful parameter for summarizing the capability of passive samplers to yield time-integrated concentrations.

2 $$\text{TIW}=\frac{{mK}_{\text{sw}}}{{R}_{\text{s}}}=\frac{{mK}_{\text{sw}}}{{Ak}_{\text{o}}}$$

where *k*_o_ is the overall mass transfer coefficient, and *A* is the water-sampler exchange surface area. Sampling is essentially time-integrative when the exposure time is smaller than the TIW (Booij et al. 2007). The TIW is often multiplied by -ln(0.5) ≈ 0.7 to obtain the half-life time for equilibrium attainment, but sampler-water equilibrium is only defined when *C*_w_ is constant, and the factor 0.7 is of minor importance. Sampler designs can be manipulated to yield larger TIWs by choosing sorbents with higher *K*_sw_, adding diffusion-limiting membranes that result in a smaller *k*_o_, and using a higher ratio of sorbent mass to surface area (Eq. 2). The use of diffusion-limiting membranes often results in lag times that originate from sorption of the target compounds to the membrane (Endo and Matsuura 2018; Vermeirssen et al. 2012).

The *R*_s_ model has also been used to model passive sampler response to peak concentration events, by evaluating if the *R*_s_ that is determined under constant *C*_w_ scenarios predicts the time-average *C*_w_ from repeated grab sampling (Bernard et al. 2018; Mazzella et al. 2008; Noro et al. 2019; Schreiner et al. 2020; Shaw and Mueller 2009). The overall conclusion from these studies is that the *C*_w_ from passive sampling and from repeated grab sampling agree within a factor of approximately 1.5, and that the presence of a polyethersulfone (PES) membrane improves the time-integrative capability of the sampler (Shaw and Mueller 2009).

Vermeirssen et al. (2012) used an empirical rate constant model for hydrophilic compound accumulation by POCIS and Chemcatchers. These authors then converted the rate constants to an equivalent *R*_s_ over an arbitrary time period of 30 d, using numerical methods. Schreiner et al. (2020) used a similar approach for Chemcatchers without a PES membrane. Analytical models for sampler-water exchange kinetics with time-variable *C*_w_ conditions have been missing so far, with the exception of the relatively simple case of *C*_w_ that varies linearly with time (Booij et al. 2003).

Tcaciuc et al. (2015) proposed to abandon *R*_s_ models because of their weak mechanistic basis, and to use diffusion models instead. These models are solutions of Fick’s second law of diffusion in the sorbent and allow modelling of partial rate control by sorbent, membrane (if present), and water boundary layer (WBL) (Booij 2021; Tcaciuc et al. 2015; Thompson et al. 2015). Analytical solutions of Fick’s second law have been applied in passive sampling research for constant *C*_w_ scenarios in infinite water volumes, and constant amount scenarios in finite water volumes.

Two approaches are available for applying diffusion models for scenarios with time-variable *C*_w_ and mixed rate control by sorbent, WBL, and membrane. Endo et al. (2019) used numerical integration of Fick’s second law for samplers with a PES membrane and a sorption phase of styrene–divinylbenzene reversed phase sulfonated polymers (SDB-RPS). Mikhailov and Özişik (1984, chapter 7.2) presented a series solution model that can be used for diffusive transport in a passive sampler sorbent, under conditions of time-variable *C*_w_ and partial rate control by the sorbent, but this model has not been applied so far in passive sampling research.

A present research challenge for passive sampling of hydrophilic compounds is to evaluate passive samplers for their capability of yielding time-integrated *C*_w_ estimates for realistic *C*_w_ scenarios, using sound mechanistic models. The aim of the present study was to assess passive sampler response to peak concentration events under semi-field conditions, to understand the effects of flow, biofouling, and presence/absence of a PES membrane on the exchange kinetics, and to evaluate the merits of a diffusion model and an *R*_s_ model for sampler-water exchange kinetics under time-variable *C*_w_. The present study was carried out in three experimental phases (Table 1). The aim of Phase 1 was to evaluate uptake and release kinetics of silicone and SDB-RPS samplers, and to study the effect of flow velocity on the exchange kinetics of SDB-RPS samplers. The purpose of Phase 2 was to evaluate sampler response to three peak concentration events, including an evaluation of flow effects. Phase 3 aimed to study differences in exchange kinetics between SDB-RPS samplers with and without a PES membrane, and to evaluate the effect of biofouling on the exchange kinetics.

**Table 1:** Summary of sampler deployment and retrieval. Spiking of Channel A was done at *t* = 1 day for Phase 1 and 3, and at *t* = 1, 3, and 6 days for Phase 2. *U* is the water flow velocity

Scenario	Treatment
Phase 1	Spike Channel A at *t* = 1 day
ChA	Deploy silicone and SDB-RPS samplers in Channel A at *U* = 9 cm/s at *t* = 0. Retrieve at regular time intervals up to 13 days
ChA- > ChB	Deploy silicone and SDB-RPS samplers in Channel A at *U* = 9 cm/s at *t* = 0
	Transfer a subset of samplers from Channel A to Channel B at *t* = 2 days. Retrieve samplers up to 13 days
Velocity effect I	*U* = 1 cm/s: deploy SDB-RPS samplers in Channel A at *U* = 1 cm/s. Transfer a subset to Channel B at *t* = 2 days. Retrieve at *t* = 8 days
	*U* = 18 cm/s: same procedure as for *U* = 1 cm/s
	*U* = 9 cm/s: use data from scenario ChA
3 x [ChA <—> ChB]	Deploy silicone and SDB-RPS samplers in Channel A at *t* = 0, *U* = 9 cm/s
	Transfer samplers to Channel B at *t* = 2 days, and back to Channel A at t = 3 days
	Transfer samplers to Channel B at *t* = 4 days, and back to Channel A at t = 5 days
	Transfer samplers to Channel B at *t* = 6 days. Retrieve at *t* = 7 days
Phase 2	Spike Channel A at *t* = 1, 3, and 6 days
ChA	Deploy silicone and SDB-RPS samplers in Channel A at *t* = 0, *U* = 9 cm/s
	Decrease concentrations by partially draining/refilling Channel A after spiking
	Retrieve up to 17 days
Velocity effect II	*U* = 1 cm/s: Deploy silicone and SDB-RPS samplers in Channel A, *U* = 1 cm/s. Retrieve samplers at *t* = 8 days,
	*U* = 18 cm/s: same procedure as for *U* = 1 cm/s
	*U* = 9 cm/s: use data from scenario ChA
Phase 3	Spike Channel A at *t* = 1 day
ChA	Deploy SDB-RPS samplers with and without PES membrane in Channel A at *t* = 0, *U* = 9 cm/s. Retrieve up to 18 days
ChA- > ChB	Deploy SDB-RPS samplers with and without PES membrane in Channel A at *t* = 0, *U* = 9 cm/s
	Transfer samplers to Channel B at *t* = 2 days. Retrieve up to 18 days
Fouling effect	Allow biofilm to develop on SDB-RPS samplers in Channel C for 2 weeks, and 4 weeks
	Deploy in Channel A at *t* = 0, *U* = 9 cm/s. Retrieve at *t* = 2 and 4 days

## Materials and methods

### Materials

Translucent silicone (Specialty Silicone Products, SSP M823, thickness 250 µm) was obtained from Shielding Solutions, UK. Sheets were cut to a size of 55 × 90 mm (mass 1.35 g, exchange surface area 0.99 dm^2^) and were pre-extracted with ethyl acetate by soaking in solvent for 1 week, followed by 12 times 90 min microwave extraction with solvent replacement (Becker et al. 2020). The sheets were then transferred to a flask with methanol for 2 days, with solvent replacement after 1 day, followed by rinsing with bi-distilled water, drying with lint-free tissue, and storage at − 20 °C.

SDB-RPS extraction disks (thickness 660 µm, disk mass 314 mg, diameter 47 mm) were obtained from Affinisep, France. Ratios of porosity (*φ*) and squared tortuosity (*θ*) were measured as *φ*/*θ *^2^ = 0.34, using the alabaster dissolution rate method (Booij et al. 2017). Disks were pre-extracted three times, by swirling gently with 20 mL methanol, followed by a 30 min rest period. Methanol was poured off and the process was repeated twice with distilled water. Disks were clamped between two stainless steel plates (bottom plate 100 × 70 mm, thickness 2 mm, upper plate 70 × 70 mm, thickness 2 mm, with a 40 mm diameter opening in the middle) (Figure S1-1). The effective sorbent mass was taken to be 314 mg × (40 mm/47 mm)^2^ = 227 mg. The exposed surface area was 0.126 dm^2^.

PES microporous membranes (Pall, Supor-200) had a thickness of 150 µm, nominal pore size of 0.2 µm, and diameter of 47 mm, and *φ*/*θ *^2^ = 0.78 (Booij et al. 2017).

### Exposure channels

Experiments were carried out in three channels from the artificial stream and pond system of the German Federal Environment Agency in Berlin-Marienfelde. Each channel consisted of two rows of 16 segments of 3 m length that were connected at the ends by U-turns, creating a closed loop with length of 104 m, width of 0.98 m, and water depth of 0.3 m (Figure S1-2). The channels were filled with water from a storage pond fed with manganese- and iron-free groundwater. The water volume in each channel was 31.35 m^3^. Screw pumps generated a flow of 9 cm/s, as measured with a Schiltknecht MiniWater 20, equipped with a MC20 MiniController and a MiniWater20 Micro sensor. Flow velocities of 18 and 1 cm/s were obtained by locally reducing channel width to 45 cm, and by inserting a secondary channel with an entrance grid, respectively (Figure S1-3). The channels contained a sediment layer of 15 cm thickness. Sediments in channel A and C were aged in the channel system over several years and can be regarded as largely natural in terms of carbon content and biology. Four segments contained macrophytes. Vegetation that had spread in other segments was removed before start of the experiments. Channel B was filled with fresh sediment and contained no plants.

Channel A was used for exposing passive samplers to elevated compound concentrations. Channel B was used for exposures in unspiked water. Channel C was used as a control channel. Peak concentration events were simulated by either transferring samplers back and forth between Channels A and B, or by partially draining Channel A followed by refilling with unspiked water.

Spiking of Channel A was done by injecting a spike solution (2 × 1 L) in methanol/water (10/90 v:v) at two opposite locations in the channel, using a valveless rotary piston pump (Ismatec, Switzerland) over a time period of 8 min. This resulted in a nearly homogeneous initial distribution of compounds over the length of the channel because spiked water travelled a distance of 43 m downstream of each spiking location during 8 min, as compared with 2 × 52 m for a complete water circulation. Spiked compounds were bentazon, carbamazepine, diclofenac, flufenacet, imidacloprid, metazachlor, nicosulfuron, pendimethalin, propiconazole, terbuthylazine, thiacloprid, and UV 326 (bumetrizole). Nominal concentrations (injected amount divided by channel volume) ranged between 50 and 1000 ng/L.

### Sampler exposures

Exposure experiments were done during three time periods in 2019: 25 June–8 July (Phase 1), 27 August–13 September (Phase 2), and 14 October–1 November (Phase 3). Sampler types were silicone and SDB-RPS extraction disks with and without a PES membrane. Sampler types and exposure scenarios are summarized below and in Table 1.

Phase 1 was designed to allow a comparison of SDB-RPS and silicone sampler response to constant *C*_w_ and to peak concentration events. Exposure to constant *C*_w_ was only successful for nicosulfuron and terbuthylazine. The *C*_w_ of other compounds decreased with time, due to a variety of loss processes (e.g., sorption to channel components, biodegradation, photodegradation). Intentional peak concentration events were simulated by transferring samplers between the spiked Channel A to the unspiked Channel B. Samplers were mounted on racks that could be quickly transferred between the channels whenever needed (Figure S1-1).

Peak concentration events during Phase 2 were generated by spiking Channel A at *t* = 1, 3, and 6 days. Immediately after each spiking event, 15 m^3^ water was drained during 35 min, followed by refilling the channel at 31 L/min for 8 h. This draining/refilling operation was repeated two times more, with a refilling rate of 25 L/min for 10 h. Total time between start of the spiking and end of the draining/refilling procedure was 1.2 days. SDB-RPS and silicone samplers were deployed during Phase 2.

Exposure scenarios during Phase 3 included sampler exposure in Channel A only and transfer to Channel B after initial exposure in Channel A. SDB-RPS samplers with and without a PES membrane were deployed. The effect of fouling was evaluated by exposing SDB-RPS samplers that had developed a biofilm during 2 and 4 weeks in Channel C.

Daily averaged temperatures decreased by approximately 13 °C during each Phase. Average temperatures were 21 °C (Phase 1, 29 to 16 °C), 19 °C (Phase 2, 25 to 14 °C), and 11 °C (Phase 3, 16 to 1 °C).

### Chemical analysis

Silicone sheets were extracted in a Soxhlet extractor (30 h, 30 mL methanol). SDB-RPS disks were shaken 3 times with 6 mL solvent (acetone, methanol, acetone) on a shaking Table (60 min, 120 RPM). Extracts were evaporated to 4 mL with a nitrogen stream. A 1 mL subsample was filtered through a PTFE filter (0.45 µm pore size), mixed with deuterated internal standards, and diluted with ultrapure water at a ratio of 1:10, 1:20, or 1:100 (v/v). Water samples were filtered and directly injected. For each batch of samples, an additional procedural blank was extracted to verify that the silicone sheets and SDB-RPS discs were not contaminated.

LC–MS/MS analysis was performed on an Agilent 1260 LC system coupled with a Sciex 4500 QTrap MS/MS System. Compounds were separated on a Phenomenex 100 × 2.1 mm Kinetex C18 column with 2.6 µm particle size and upstream security guard cartridge at 40 °C, using a gradient of 0.4 mM ammonium acetate in water and methanol. The tandem mass spectrometer was run in multiple reaction mode (MRM) with positive and negative electrospray ionization. Details of instrumental analysis, internal standards, detection limits, and quantitation limits are given in section S2.

### Modelling of aqueous concentrations

Concentrations in water were modelled as an exponential decrease from the value immediately after spiking (*C*_w0_) towards a constant level (*C*_w∞_) as *t→∞*

3 $${C}_{\text{w}}=\left({C}_{\text{w}0}-{C}_{\text{w}\infty }\right)\text{exp}\left(-\alpha t\right)+{C}_{\text{w}\infty }$$

where α is a first-order decay constant and *t* is the time after spiking.

Accuracy of the *C*_w_ data was evaluated from the mass balance (*MB*) at the time of spiking.

4 $$MB=\frac{{C}_{\text{w}0}}{{C}_{\text{w},\text{pre}-\text{spike}}+{C}_{\text{w}0,\text{spike}}}\times 100\%$$

where (*C*_w,pre-spike_) is the concentration immediately before spiking and *C*_w,0,spike_ is the increase in *C*_w_, as calculated from spiked amount and channel volume.

Modelled *C*_w_ values (Eq. 3) were used for evaluating sampler-water exchange kinetics for all post-spike periods. Measured aqueous concentrations > LOQ were adopted for Channel B and for the pre-spike period in Channel A, but some inevitable uncertainty is associated with *C*_w_ values < LOQ, as *C*_w_ can have any value between 0 and LOQ in these cases. We chose for a conservative approach for dealing with this uncertainty. Concentrations below the LOQ were set to zero because the exposure water was aged, and isolated from direct and indirect sources. In some exceptional cases, there was evidence that *C*_w_ was > 0, as measurable amounts were detected in the samplers before the first spiking event. This was observed in Phase 2 for metazachlor, and in Phase 3 for metazachlor, nicosulfuron, and thiacloprid. After initial modelling of sampler-water exchange kinetics the pre-spike *C*_w_ of these compounds was adjusted to match the amounts in the samplers before spiking. For Channel B, metazachlor concentrations of 0.005 ng/mL were adopted because amounts in the samplers reached a plateau value in this channel during Phases 1 and 3. We verified that all hypothesized *C*_w_ values were indeed < LOQ.

### Sampling rate model with time-variable *C*_*w*_

The *R*_s_ model can be generalized to account for time-dependent *C*_w_ and nonzero initial concentrations (supplementary information, S3)

5 $${C}_{\text{s}}\left(t\right)=\text{exp}\left(-{k}_{\text{e}}t\right)\left({C}_{\text{s}0}+{k}_{\text{e}}{K}_{\text{sw}}\underset{0}{\overset{t}{\int }}\text{exp}\left({k}_{\text{e}}{t}^{\prime}\right){C}_{\text{w}}\left({t}^{\prime}\right){dt}^{\prime}\right)$$

where *C*_s0_ is the initial concentration in the sampler, and *k*_e_ = *R*_s_/(*mK*_sw_) is used to simplify notation. Evaluation of the integral is straightforward for the adopted *C*_w_ model (Eq. 3). The time domain was divided in separate stages (pre-spike and post-spike periods), each starting with its own *t* = 0. The final *C*_s_ from the previous stage was used as the *C*_s0_ of the next stage. The most complex scenario was repeated sampler switching between Channels A and B during Phase 1, which comprised 7 stages (pre-spike exposure + three periods in Channel A + three periods in Channel B).

Combining the *C*_w_ model (Eq. 3) and the generalized *R*_s_ model (Eq. 5) yields the *R*_s_ model for exposures in Channel A.

6 $${C}_{\text{s}}\left(t\right)={K}_{\text{sw}}{C}_{\text{w}\infty }+\left({C}_{\text{s}0}-{K}_{\text{sw}}{C}_{\text{w}\infty }\right)\text{exp}\left(-{k}_{\text{e}}t\right)+{k}_{\text{e}}{K}_{\text{sw}}\left({C}_{\text{w}0}-{C}_{\text{w}\infty }\right)\frac{\text{exp}\left(-\alpha t\right)-\text{exp}\left(-{k}_{\text{e}}t\right)}{{k}_{\text{e}}-\alpha }$$

Exposures at constant *C*_w_ were modeled by setting *C*_w0_ = *C*_w∞_. For nonlinear least squares analysis *k*_e_ was replaced by *R*_s_/(*mK*_sw_), because optimizing *K*_sw_ and *k*_e_ would yield erroneous estimates of the standard errors of *R*_s_.

### Diffusion model with time-variable *C*_*w*_

The diffusion model from Mikhailov and Özişik (1984) is more compactly written in terms of dimensionless time (*τ*)

7 $$\tau =\frac{{D}_{\text{s}}t}{{L}^{2}}$$

where *L* is the half-thickness for samplers that are exposed on both sides (silicone in the present study) or the thickness for samplers that are exposed on one-side, with an impermeable boundary at the other side (SDB-RPS in the present study). Model derivation for plane sheets is summarized in section S4. The time evolution of space-averaged concentrations is given by

8 $${C}_{\text{s}}\left(\tau \right)=\sum_{n=1}^{\infty }\frac{\text{sin}({\mu }_{\text{n}})}{{F}_{\text{n}}{\mu }_{\text{n}}}{e}^{-{\mu }_{\text{n}}^{2}\tau }\left[{\widetilde{C}}_{\text{s},\text{n}}\left(0\right)+{\mu }_{\text{n}}\text{sin}({\mu }_{\text{n}}){K}_{\text{sw}}^{\prime}\underset{0}{\overset{\tau }{\int }}{C}_{\text{w}}({\tau }^{\prime}){e}^{{\mu }_{\text{n}}^{2}{\tau }^{\prime}}{d\tau }^{\prime}\right]$$

where *µ*_n_ are solutions of

9 $$\text{cot}\left({\mu }_{\text{n}}\right)-\frac{{\mu }_{\text{n}}}{Bi}=0$$

The Biot number (Bi) is defined as

10 $$Bi=\frac{{k}_{\text{w}}L}{{{K}^{\prime}}_{\text{sw}}{D}_{\text{s}}}$$

The term *F*_n_ in the denominator of Eq. 8 is a normalization factor.

11 $${F}_{\text{n}}=\underset{0}{\overset{1}{\int }}{\text{cos}}^{2}\left({\mu }_{\text{n}}X\right)dX=\frac{1}{2}+\frac{\text{cos}\left({\mu }_{\text{n}}\right)\text{sin}({\mu }_{\text{n}})}{{2\mu }_{\text{n}}}$$

where *X* = *x*/*L*. The term ${\widetilde{C}}_{\text{s},\text{n}}(0)$ between the square brackets in Eq. 8 is the integral transform of the initial concentration in the sorbent

12 $${\widetilde{C}}_{\text{s},\text{n}}\left(0\right)=\underset{0}{\overset{1}{\int }}\text{cos}({\mu }_{\text{n}}X){C}_{\text{s}}(X,0)dX$$

Substituting the *C*_w_ model (Eq. 3) into Eq. 8 yields

13 $${C}_{\text{s}}\left(\tau \right)=\sum_{n=1}^{\infty }\frac{\text{sin}\left({\mu }_{\text{n}}\right)}{{F}_{\text{n}}{\mu }_{\text{n}}}\left[{\widetilde{C}}_{\text{s},\text{n}}\left(0\right){e}^{-{\mu }_{\text{n}}^{2}\tau }+{\mu }_{\text{n}}\text{sin}\left({\mu }_{\text{n}}\right){K}_{\text{sw}}^{\prime}\left\{\frac{{C}_{\text{w}0}-{C}_{\text{w}\infty }}{-\beta +{\mu }_{\text{n}}^{2}}\left({e}^{-\beta \tau }-{e}^{-{\mu }_{\text{n}}^{2}\tau }\right)+\frac{{C}_{\text{w}\infty }}{{\mu }_{\text{n}}^{2}}\left(1-{e}^{-{\mu }_{\text{n}}^{2}\tau }\right)\right\}\right]$$

where *β* = α*L*^2^/*D*_s_. Constant-*C*_w_ scenarios are covered by setting *C*_w0_ = *C*_w∞_.

The diffusion model has *K*’_sw_, *D*_s_, and *k*_w_ as adjustable parameters. For diffusion in porous media (SDB-RPS) this can be reduced to two parameters because *D*_s_ is inversely proportional to *K*’_sw_ by (Booij 2021; Endo et al. 2019)

14 $${D}_{\text{s}}=\frac{{\varphi }_{\text{s}}{D}_{\text{w}}}{{\theta }_{\text{s}}^{2}{K}_{\text{sw}}^{\prime}}$$

*D*_w_ at 25 °C was calculated from the McGowan molar volume (Schwarzenbach et al. 2016), and correction for exposure temperature was done using the relationship with dynamic viscosity (*D*_w_ ~ *η*^−1.14^) as suggested by Hayduk and Laudie (1974).

The time domain was divided into multiple stages that started at the time of sudden change in *C*_w_ (a new spike event or a transfer between Channels A and B), as outlined for the *R*_s_ model above. The initial concentration distribution for each next stage was set equal to the final concentration distribution for the previous stage.

### Series resistance model

The series resistance model is useful for understanding the rate limiting steps and the effects of temperature, flow, and biofouling on the exchange kinetics. For single-phase polymers without biofouling layer this model takes the form (Booij 2021)

15 $$\frac{1}{{k}_{\text{o}}}=\frac{1}{{k}_{\text{w}}}+\frac{{\delta }_{\text{s}}(t)}{{K}_{\text{sw}}^{\prime}{D}_{s}}$$

where *k*_o_ is the overall mass transfer coefficient (i.e., the proportionality constant between the flux and the effective concentration difference between sampler and water), *k*_w_ is the mass transfer coefficient of the WBL, and *D*_s_ is the diffusion coefficient in the sorbent. The *K*’_sw_ is the sorption coefficient in volume per volume units (concentration in bulk sorbent divided by concentration in water). The *δ*_s_(*t*) is the effective thickness of the concentration boundary layer in the sorbent. The concentration gradient in the sorbent is infinite at *t* = 0, and hence *δ*_s_(0) = 0. At increasing time, *δ*_s_(*t*) gradually increases, and reaches a steady state value of approximately 0.3 to 0.4 times the half-thickness (*L*) when *D*_s_*t*/*L*^2^ > 0.1.

For porous sorbents, porous membranes, and biofouling, present knowledge suggests that the diffusion coefficient is inversely proportional to the sorption coefficient, which causes the product *K*’_sw_*D*_s_ to be independent of *K*’_sw_ (Booij 2021; Booij et al. 2007)

16 $$\frac{1}{{k}_{\text{o}}}=\frac{1}{{k}_{\text{w}}}+\frac{{\theta }_{\text{m}}^{2}{d}_{\text{m}}}{{\varphi }_{\text{m}}{D}_{\text{w}}}+\frac{{\theta }_{\text{s}}^{2}{\delta }_{\text{s}}(t)}{{\varphi }_{\text{s}}{D}_{\text{w}}}+\frac{{\theta }_{\text{b}}^{2}{d}_{\text{b}}}{{\varphi }_{\text{b}}{D}_{\text{w}}}$$

where subscripts m and b refer to membrane and biofilm, *ϕ* is porosity, *θ* is tortuosity, and *d* is the thickness of membrane or biofilm. Here it is assumed that transport in membrane and biofilm is through the pore space only. The sorption coefficient *K*’_sw_ still occurs implicitly in *δ*_s_(*t*), because the concentration gradient in the sorbent is steeper for longer times when *K*’_sw_ is larger.

### Statistical methods

Best-fit parameters and their standard errors were obtained using weighted nonlinear least squares analysis in Microsoft Excel, using the Solver add-in (Billo 2001). A clear minimum in the residual sum of squares (SSQ) was not always observed. For the diffusion model this can occur when the kinetics is essentially controlled by the sorbent. A plot of SSQ vs. *k*_w_ shows a plateau at large *k*_w_ in that case, and only rises for *k*_w_ values that are too small. The nature of the SSQ minimum (local minimum, global minimum, or plateau) was evaluated by running the model at fixed *k*_w_ values between 0.1 and 1000 µm/s, optimizing the other parameters, and plotting SSQ vs. *k*_w_. When a plateau was found in the SSQ vs. *k*_w_ plot, it was concluded that the simpler sorbent control model was applicable. When a minimum was found in the SSQ vs. *k*_w_ plot, a partial *F*-test was used to determine if the complex model (adjustable *k*_w_) yielded a significantly better fit than the reduced model (fixed *k*_w_ = 1000 µm/s).

17 $$F=\frac{\frac{{SSQ}_{\text{reduced}}-{SSQ}_{\text{complex}}}{{df}_{\text{reduced}}-{df}_{\text{complex}}}}{\frac{{SSQ}_{\text{complex}}}{{df}_{\text{complex}}}}$$

Values of *k*_w_ were labelled as not significant when the *F*-value was smaller than the critical *F*-value (95% level of confidence, right-tailed *F*-test).

Similarly, for WBL-controlled kinetics, any *D*_s_ value that is larger than a certain threshold gives an equally good fit. Finally, when *C*_s_ is much smaller than the equilibrium concentration, then any high value of *K*_sw_ or *K*’_sw_ will do. The significance of *D*_s_ and *K*_sw_ or *K*’_sw_ was evaluated similarly to the significance of *k*_w_. The approach is further illustrated in section S5.

The partial *F*-test was also used to determine if statistically significant lag times occurred in the *R*_s_ model for SDB-RPS samplers that were covered by a PES membrane.

## Results and discussion

### Aqueous concentration modelling and compound selection

Modelling of *C*_w_ in Channel A was considered to be acceptable when a sufficient number of *C*_w_ values was above the LOQ, and when the mass balance at the time of spiking was between 80 and 120%. Bentazon, carbamazepine, imidacloprid, metazachlor, nicosulfuron (Phase 1 and 3 only), terbuthylazine (Phase 1 and 2 only), and thiacloprid, passed these criteria (Table 2). Mass balances for nicosulfuron during Phase 2 (120 to 160%) and for terbuthylazine in Phase 3 (57%) were outside the 80 to 120% window, but were still retained to allow comparison of sampler kinetics among the respective experimental Phases. *C*_w_ data for diclofenac, flufenacet, pendimethalin, propiconazole, and UV 326 did not meet the criteria for mass balance and detection frequency in any of the experimental Phases, and were excluded from further consideration (Tables S6-1, S6-2, and S6-3). The rapid disappearance of pendimethalin and UV 326 may be related to sorption to channel walls and sediment sections because of their relatively high log*K*_ow_ values of 5.20 and 5.55. The rapid disappearance of diclofenac may be related to degradation. The reason for low mass balances of flufenacet and propiconazole is unclear.

**Table 2:** Parameters for aqueous concentration modelling of included compounds. (Model results for excluded compounds are given in Tables S6-1, S6-2, and S6-3)

	Phase 1			Phase 2					Phase 3		
Compound	Mass balance	*α*	*n*	Mass balance peak 1	Mass balance peak 2	Mass balance peak 3	*α*	*n*	Mass balance	*α*	*n*
	(%)	(day^−1^)		(%)	(%)	(%)	(day^−1^)		(%)	(day^−1^)	
Bentazon	93	0.096	65	121	100	96	0.98	7	85	0.027	16
Carbamazepine	82	0.017	65	96	100	98	2.3	32	97	0.56	16
Imidacloprid	82	0.64	56	94	132	97	2.1	18	92	0.14	16
Metazachlor	91	0.035	65	105	100	101	1.9	32	102	0.51	16
Nicosulfuron	103	0	65	160	148	121	1.7	9	107	0	16
Terbuthylazine	82	0	65	104	87	90	2.0	32	57	0	16
Thiacloprid	80	0.028	65	94	95	92	1.9	29	87	0.043	16

### Silicone samplers

**Fig. 1:**
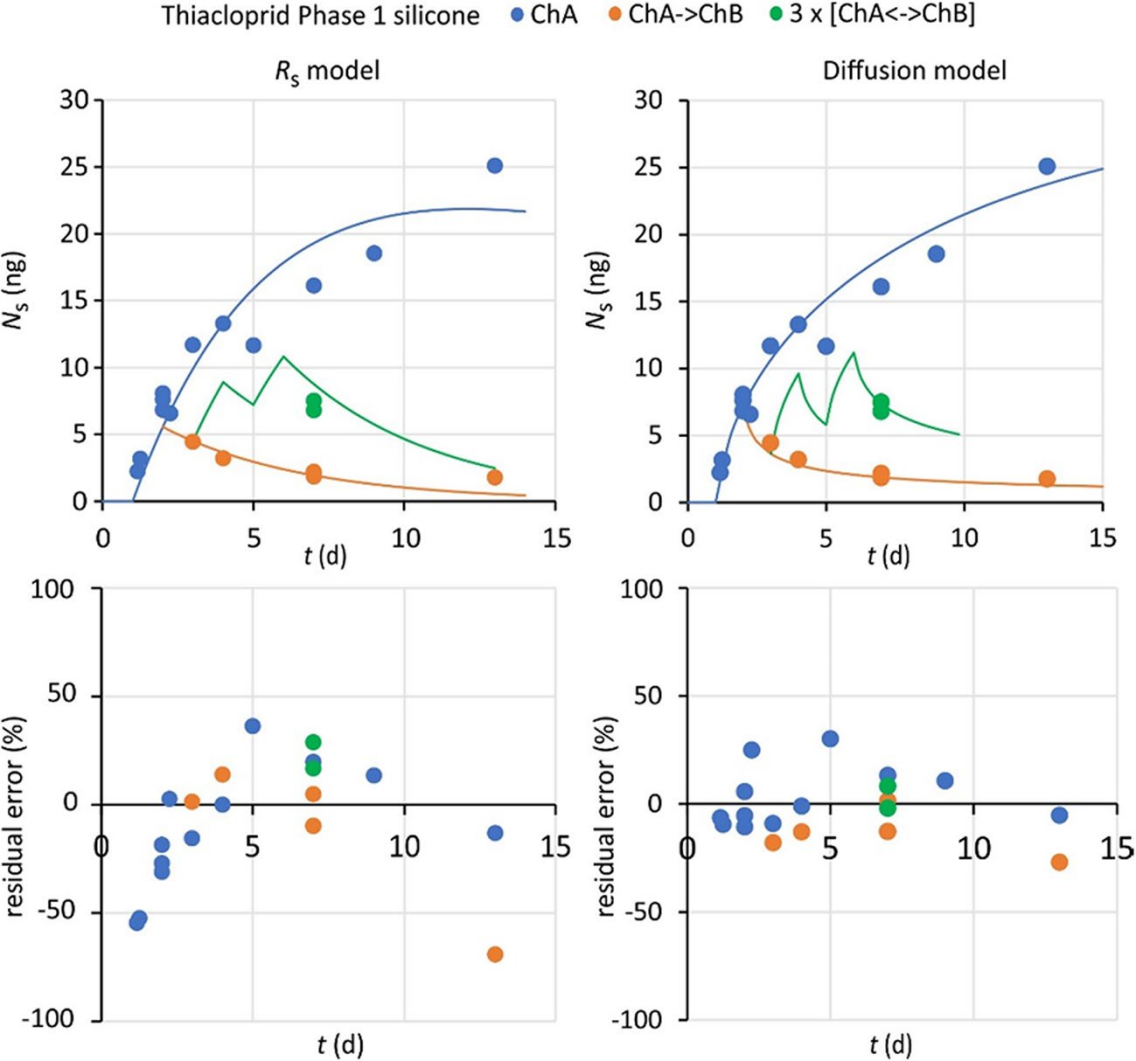
Model fit and residual errors of thiacloprid sampling by silicone samplers in Phase 1 for the *R*_s_ model (left) and the diffusion model (right). *C*_w_ exposure scenarios were continuous exposure in Channel A (blue), exposure in Channel A for 2 days, followed by transfer to Channel B (amber), and three times switch between Channels A and B (green). Solid lines represent model fits

No accumulation in silicone was observed for bentazon, imidacloprid, and nicosulfuron. Accumulation of carbamazepine, metazachlor, terbuthylazine, and thiacloprid is characterized by low sorption coefficients (log*K*_sw_ between 2.0 and 3.4) and large differences in *R*_s_/A between compounds (0.1 to 33 L/[dm^2^ day])(Table 3). Amounts that are accumulated during a peak concentration event dissipate appreciable within a few days (Fig. 1, and Figure S7-1). TIW values are between 0.5 and 6 days, which makes silicone to be of little practical use for time-integrative sampling of these compounds, although this sampler is still interesting for research purposes.

**Table 3:** Model results for silicone samplers. SE = standard error of the parameter in the column to the left. No accumulation was observed for bentazon, imidacloprid, and nicosulfuron. TIW is the time-integrative window (*mK*_sw_/*R*_s_)

		Diffusion model								*R*_s_ model					
		log*K*'_sw_ (mL/mL)	SE	*D*_s_ (µm^2^/s)	SE	*k*_w_ (µm/s)	SE	*s* (%)	*n*	log*K*_sw_ (mL/g)	SE	*R*_s_/*A* (L/[dm^2^ day])	SE	*s* (%)	TIW (days)
Carbamazepine	Phase 1	2.26	0.13	0.0056	0.0038	ns)^1^	-	16	24	2.04	0.04	0.32	0.05	40	3.7
	Phase 2	2.28	0.01	0.0088	0.0010	0.4	0.2	13	25	2.13	0.03	0.36	0.04	19	4.0
Metazachlor	Phase 1	2.88	0.02	0.15	0.04	7.2	3.1	21	24	2.87	0.02	15	1	24	0.5
	Phase 2	3.10	0.06	0.012	0.004	ns)^1^	-	28	25	2.94	0.03	8.6	2.0	33	1.1
Terbuthylazine	Phase 1	3.34	0.05	0.080	0.051	ns)^1^	-	18	24	3.30	0.02	33	5	20	0.6
	Phase 2	3.48	0.02	0.032	0.007	ns)^1^	-	19	25	3.44	0.02	24	4	19	1.2
Thiacloprid	Phase 1	3.10	0.18	0.000029	0.000020	ns)^1^	-	15	20	1.74	0.05	0.12	0.02	30	4.7
	Phase 2	2.97	0.21	0.000044	0.000038	ns)^1^	-	20	25	1.76	0.06	0.10	0.01	26	6.0

)^1^ Including *k*_w_ as an adjustable parameter did not yield a significantly better fit

**Fig. 2:**
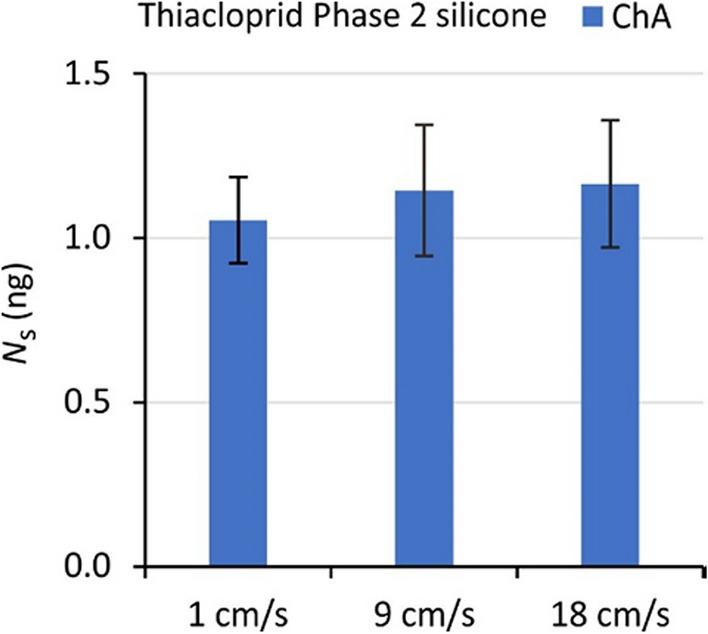
Accumulation of thiacloprid by silicone samplers in Phase 2 for three flow velocities. Exposure in Channel A, sampling at *t* = 8 days

The diffusion model gave a better fit of the data than the *R*_s_ model (residual errors 13–28% vs. 19–40%, Table 3). No difference in residual errors was found for terbuthylazine (19%). Better performance of the diffusion model is supported by the more randomly distributed residual errors for this model (Fig. 1, Figure S7-1). No significant effect of flow (Phase 2) was observed for any of the compounds between 1 and 18 cm/s (Fig. 2, Figure S8-1).

Including *k*_w_ as an adjustable parameter in the diffusion model did not yield a better fit, except for carbamazepine in Phase 2 and metazachlor in Phase 1 (Table 3). This indicates insignificant rate control by the WBL. The *k*_w_ estimate for carbamazepine in Phase 2 (0.4 ± 0.2 µm/s) is statistically significant (*p* = 0.03), but suspiciously small and likely erroneous. Significant flow effects would be expected for this compound if the *k*_w_ estimate were realistic, but no flow effects were observed (Figure S8-1). Model results for fully sorbent-controlled kinetics yielded slightly higher residual errors (14% vs. 13%), and smaller *D*_s_ (0.0057 µm^2^/s) for carbamazepine. The *k*_w_ estimate for metazachlor in Phase 1 (7.2 ± 3.1 µm/s) is significant (*p* = 0.02) and realistic, but no flow effects were observed for this compound for the Phase 2 data. We have no solid explanation for the statistical significance of *k*_w_ for these two cases, but we consider that the relatively large standard errors of approximately 50% may be taken as a warning that the *k*_w_ estimates are questionable after all. This further illustrates that conclusions on expected flow effects that are based on curve fitting need experimental verification by exposing samplers to different flow velocities.

### SDB-RPS samplers without a membrane

Sorption coefficients for SDB-RPS samplers are substantially higher than for silicone (log*K*_sw_ between 3.5 and 4.7; median values for Phases 1, 2, and 3, Table 4). *R*_s_/A values range between 0.8 and 4.0 L/[dm^2^ day] (*R*_s_ between 0.1 and 0.5 L/day). The TIWs between 7 and 34 days indicate fair time-integrative capability for exposures of two weeks, except for bentazon (TIW = 10 days) and nicosulfuron (TIW = 7 days).

**Table 4:** Model results for SDB-RPS samplers. SE = standard error of the parameter in the column to the left. The diffusion coefficient in the sorbent (*D*_s_) follows from Eq. 14 and the optimized value of *K*’_sw_. TIW is the time-integrative window (*mK*_sw_/*R*_s_)

Compound	Phase	Diffusion model						*R*_s_ model					
		log*K*'_sw_ (mL/mL)	SE	*k*_w_ (µm/s)	SE	*s* (%)	*N*	log*K*_sw_ (mL/g)	SE	*R*_s_/*A* (L/[dm^2^ day])	SE	*s* (%)	TIW (days)
Bentazon	1	3.07	0.03	6.8	1.0	17	38	3.42	0.03	1.3	0.1	35	3.5
	2	3.53	0.26	0.7	0.1	25	31	ns)^1^		0.38	0.02	27	> 16
	3	3.30	0.06	2.6	0.4	24	46	3.63	0.06	0.80	0.06	33	9.5
Carbamazepine	1	5.01	0.07	8.9	0.6	18	38	4.37	0.05	5.2	0.3	23	8.0
	2	5.08	0.08	6.6	0.6	13	36	4.43	0.05	4.0	0.2	15	12
	3	5.48	0.16	3.4	0.2	20	53	4.63	0.09	2.6	0.09	20	30
Imidacloprid	1	4.42	0.05	9.4	0.9	18	38	4.13	0.04	3.9	0.3	29	6.2
	2	4.87	0.14	3.4	0.4	15	30	4.47	0.11	2.1	0.1	17	25
	3	4.51	0.12	4.1	0.5	31	49	4.32	0.11	2.0	0.2	36	19
Metazachlor	1	5.39	0.09	7.3	0.4	16	38	4.51	0.05	5.1	0.2	17	11
	2	ns)^1^		3.9	0.2	14	36	ns)^1^		3.2	0.1	14	> 16
	3	5.23	0.15	3.8	0.3	23	53	4.45	0.08	2.7	0.1	23	19
Nicosulfuron	1	3.01	0.05	1.9	0.2	21	38	3.30	0.04	0.75	0.06	32	4.7
	2	3.17	0.13	1.9	0.2	21	38	ns)^1^	ns)^1^	0.45	0.04	32	> 16
	3	3.54	0.12	2.4	0.5	46	52	3.63	0.04	1.1	0.1	44	7.0
Terbuthylazine	1	4.99	0.09	8.7	0.8	17	38	4.30	0.04	5.2	0.2	17	6.8
	2	5.59	0.14	5.6	0.4	13	36	4.67	0.07	4.0	0.2	13	21
	3	4.94	0.11	5.6	0.4	13	36	4.29	0.05	2.9	0.1	13	12
Thiacloprid	1	5.20	0.07	7.9	0.5	16	38	4.45	0.05	5.1	0.2	19	9.9
	2	5.83	0.23	4.5	0.3	13	36	4.84	0.15	3.4	0.1	14	37
	3	5.50	0.18	3.5	0.2	22	53	4.69	0.12	2.6	0.1	22	34

)^1^ Including *K*’_sw_ or *K*_sw_ as an adjustable parameter did not yield a significantly better fit

**Fig. 3:**
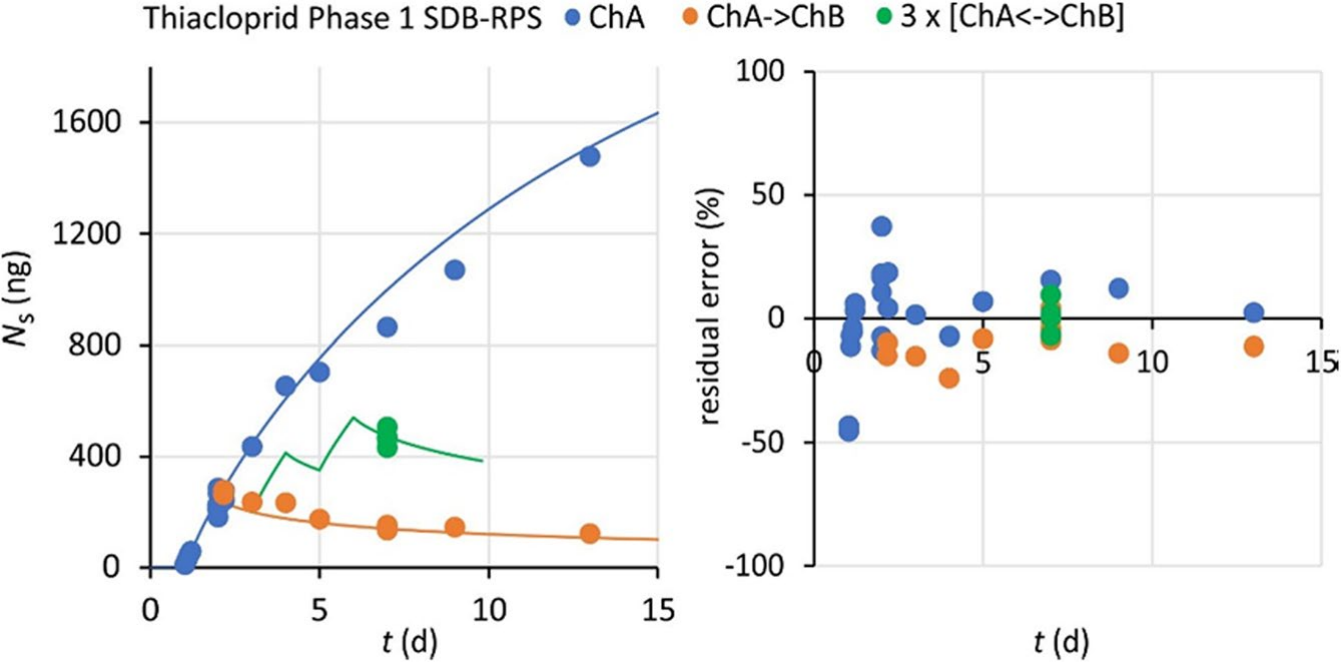
Model plot (left) and residual errors (right) for thiacloprid sampling by SDB-RPS samplers in Phase 1, analyzed with the diffusion model. Results for the *R*_s_ model are nearly identical (Figure S7-1)

The diffusion model yielded a better fit of the data for SDB-RPS samplers than the *R*_s_ model, but the difference in residual errors was typically only a few percent (Table 4). The largest difference was observed for bentazon (17 to 25% for the diffusion model versus 27 to 35% for the *R*_s_ model). The relatively large residual errors (44 to 46%) for nicosulfuron in Phase 3 result from appreciable scatter for *t* < 4 days (Figure S9-1). Residual errors were fairly randomly distributed, both for uptake in Channel A and for dissipation in Channel B (Fig. 3, Figure S9-1). This indicates that uptake and release kinetics are well-described by the same model parameter values, and that anisotropic exchange was not a major issue for the present data. Accumulated amounts with SDB-RPS are much higher than for silicone, due to the higher sorption coefficients of SDB-RPS. A similarity for these samplers is that accumulated compounds in SDB-RPS also dissipate on a time scale of a few days (Fig. 3, Figure S9-1).

**Fig. 4:**
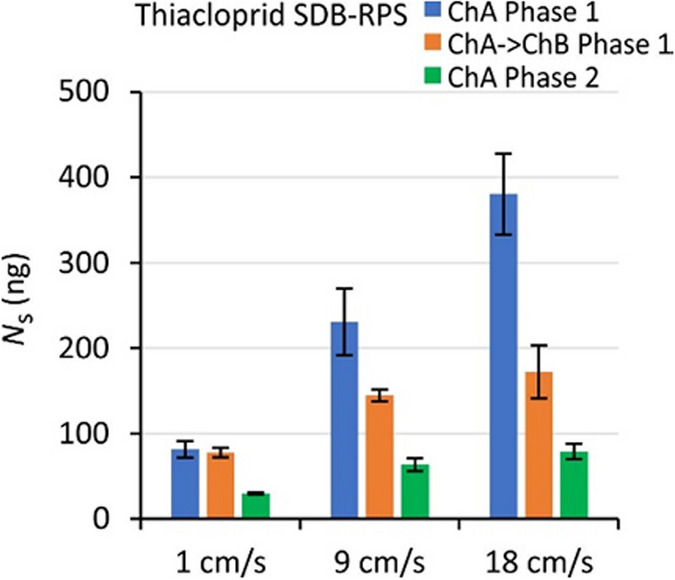
Effect of flow on the accumulated amounts of thiacloprid in SDB-RPS samplers. Error bars represent standard deviations

Amounts in SDB-RPS samplers increased with flow velocity for all exposure scenarios that were evaluated (Fig. 4, Figure S10-1). For fully WBL-controlled kinetics during the kinetic sampling stage at constant *C*_w_ it is expected that amounts follow a power law relationship with flow velocity: *N*_s_ ~ *U*^*n*^, with *n* = 1/2, because *k*_w_ increases with the square root of *U* (Glanzmann et al. 2022; Stephens et al. 2005). Experimental values of *n* (obtained from the slopes of log*N*_s_ versus log*U*) were 0.30 ± 0.15 (range 0 to 0.53, Table S10-1). The observation that slopes are generally smaller than 0.5 likely originates from partial equilibrium attainment and from the fact that the kinetics is only partially WBL-controlled.

**Fig. 5:**
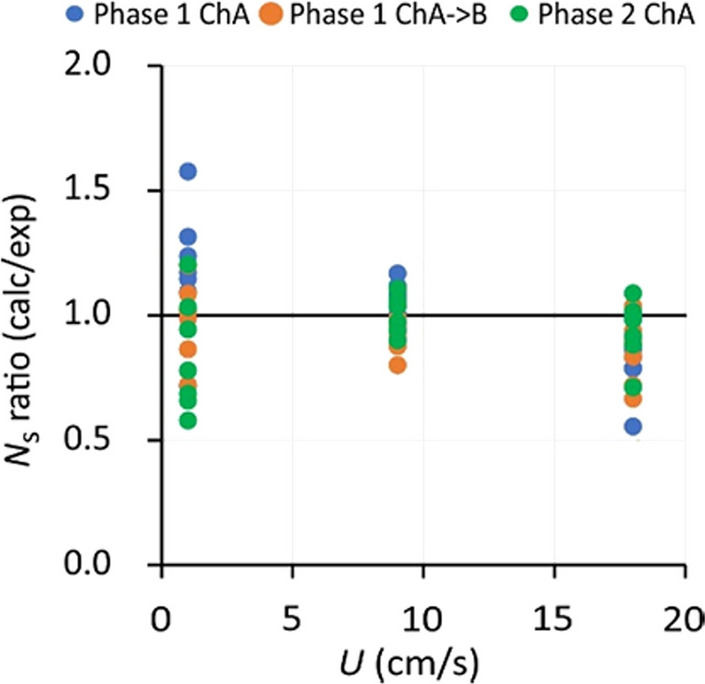
Modelling of flow effects on the accumulation in SDB-RPS samplers. Vertical axis: ratio of modelled and experimental amounts at flow velocities of 1, 9, and 18 cm/s, adopting the relationship *k*_w_ ~ *U*^1/2^

The applicability of the square root relationship between *k*_w_ and *U* was further evaluated as follows. First, the amounts at a flow velocity of 9 cm/s were calculated from the model parameters in Table 4. The amounts at *U* = 1 and 18 cm/s were then calculated by using a 3-times lower and √2 times higher *k*_w_, respectively. The ratio of calculated and measured amounts was 0.96 ± 0.18 (Fig. 5). The model slightly underestimated the amounts at 18 cm/s (amount ratio 0.87 ± 0.13), but overall gives a fair prediction of flow effects for the present velocity range.

### SDB-RPS samplers with a PES membrane

**Fig. 6:**
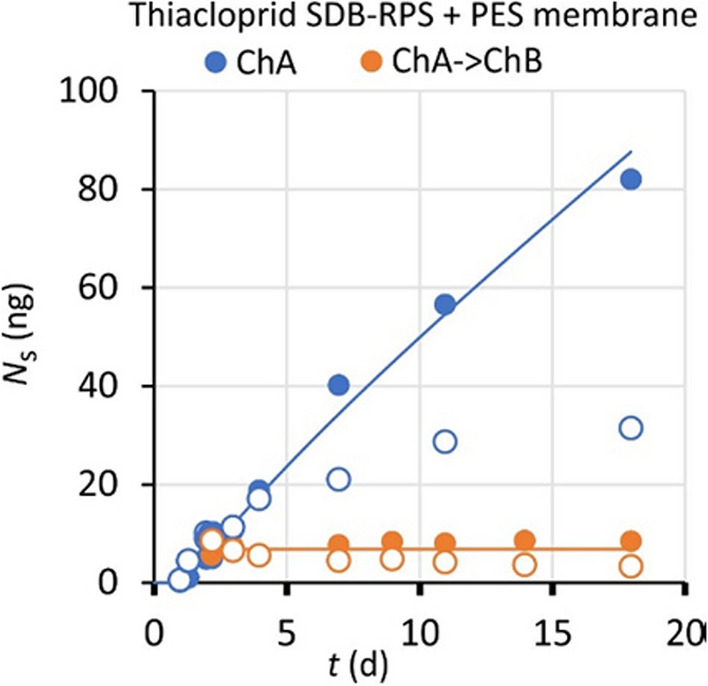
Accumulation of thiacloprid in SDB-RPS (filled circles) and PES membrane (open circles), for uptake in Channel A (blue) and dissipation in Channel B (amber)

In SDB-RPS samplers with a PES membrane appreciable sorption to PES occurred for thiacloprid and terbuthylazine (Fig. 6, Figure S11-1). Sorption of bentazon, carbamazepine, imidacloprid, and metazachlor to PES was less than 20% at the end of the exposure. Nicosulfuron showed erratic responses (residual errors of 65%, Figure S11-1), and is excluded from further discussion of membrane effects. Amounts in samplers with a membrane were 2 to 3 times smaller than the amounts in samplers without a membrane, in line with observations by Shaw et al. (2009). An exception was bentazon, for which the amounts in both sampler types were approximately the same, which indicates again that the exchange kinetics for this compound is controlled by the sorbent.

The presence of a PES membrane appreciably improved the time-integrative capabilities of SDB-RPS samplers. TIWs were between 17 and 140 days (Table 5). *R*_s_/*A* values spanned a narrower range (0.6 to 0.9 L/[dm^2^ day]) than for samplers without a membrane (0.8 to 4.0 L/[dm^2^ day]). Minor lag times were observed for carbamazepine (0.1 days), terbuthylazine (0.4 days), thiacloprid (0.2 days). For the present set of compounds, the disadvantages of analyte sorption to PES are outweighed by the advantages of higher TIWs and more consistent *R*_s_ values.

**Table 5:** *R*_s_ model results including lag times for SDB-RPS samplers with a PES membrane. SE = standard error of the parameter in the column to the left. TIW is the time-integrative window (*mK*_sw_/*R*_s_)

	log*K*_sw_ (mL/g)	SE	Lag time (days)	SE	*R*_s_/A (L/[dm^2^ day])	SE	*n*	*s* (%)	TIW (days)
Bentazon	3.78	0.05	ns)^1^		0.63	0.03	18	14	17
Carbamazepine	ns)^1^		0.06	0.03	0.87	0.03	18	11	55)^2^
Imidacloprid	ns)^1^		ns)^1^		0.71	0.03	16	15	53)^2^
Metazachlor	ns)^1^		ns)^1^		0.77	0.02	20	10	71)^2^
Terbuthylazine	4.05	0.08	0.38	0.06	0.91	0.06	15	8	22
Thiacloprid	ns)^1^	ns	0.21	0.02	0.62	0.03	17	16	140)^2^

)^1^ Including *K*_sw_ or lag time as an adjustable parameter did not yield a significantly better fit

)^2^ Based on the median log*K*_sw_ from data for SDB-RPS disks without a membrane (Table 4)

The diffusion model yielded the same residual errors as the *R*_s_ model for all compounds except bentazon (Table S11-1). Residual errors were approximately 13% for carbamazepine, imidacloprid, and metazachlor, and were much larger for terbuthylazine (26%) and thiacloprid (38%). For the latter two compounds modeled amounts at short time scales were 80% higher than measured, and up to 40% lower at long time scales (Figure S11-1). This error structure is in line with the occurrence of lag times that originate from sorption to PES. This has been observed for sampling by Chemcatchers with SDB-RPS sorbent and PES membranes, for compounds with log*K*_PES-w_ larger than 2 (Vermeirssen et al. 2012) or 3.5 (Estoppey et al. 2019). Data from Vermeirssen et al. (2012) indicate lag times for terbuthylazine of 0.5 to 4 days for Chemcatchers and 0.2 to 2 days for POCIS with HLB sorbent.

Lag times (*t*_lag_) were evaluated using the *R*_s_ model, by replacing *t* with *t*-*t*_lag_ in Eq. 6, and ignoring amounts for *t* < *t*_lag_. Including lag times in the model yields a better fit only for carbamazepine, terbuthylazine, and thiacloprid (Table 5, Figure S11-2). Lag times < 0.4 days are minor when compared with typical exposure times of 2 weeks. Residual errors are slightly smaller for samplers with a PES membrane than for bare SDB-RPS samplers (12 vs 20%). Sampling rates are similar for all compounds (*R*_s_ = 95 ± 15 mL/day; mean ± standard deviation). This value is fairly well in line with a series resistance model for mixed WBL/membrane controlled kinetics (Eq. 16). Adopting *φ*/*θ *^2^ = 0.78 for the PES membrane, *D*_w_ = 350 µm^2^/s at 11 °C during Phase 3, a membrane thickness of 150 µm, and assuming transport through the pore space only, results in a membrane conductivity of 0.78 × 350/150 = 1.8 µm/s (Eq. 16). Adopting further a WBL conductivity (*k*_w_) for Phase 3 of 3.6 µm/s (Table 4), yields a combined conductivity of (1/1.8 + 1/3.6)^−1^ = 1.2 µm/s = 1.0 L/(dm^2^ day). For a surface area of 0.126 dm^2^ the expected *R*_s_ would be 130 mL/day, which is 1.4 times higher than the experimental *R*_s_. Further understanding of the experimental *R*_s_ can be gained when experimental values of porosity and tortuosity for the present membrane type are available.

### *k*_*w*_ estimates

**Fig. 7:**
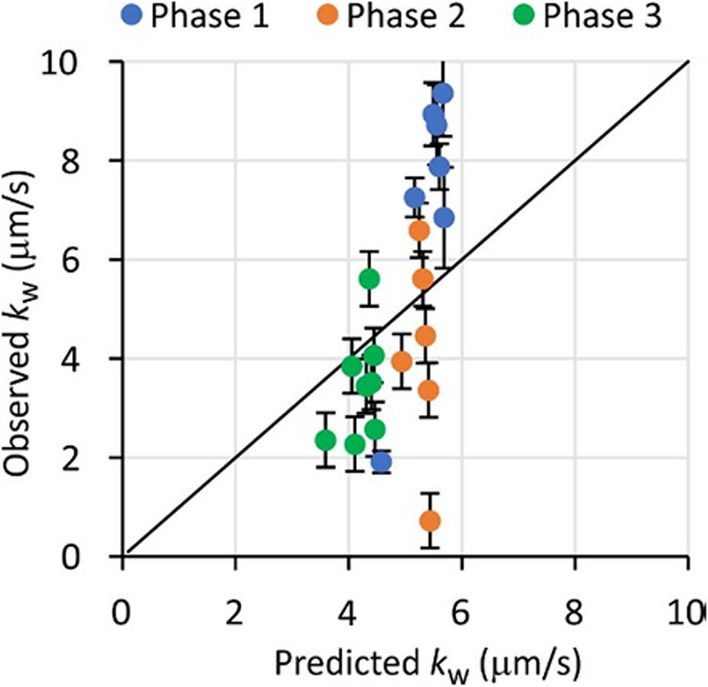
Estimates of *k*_w_ from the diffusion model versus predicted values from Glanzmann et al. (2022). The solid line represents the 1:1 relationship. Error bars represent standard errors

Experimental *k*_w_ values for SDB-RPS samplers were similar to the values that were calculated from water flow velocities and water properties, using the model from Glanzmann et al. (2022). (Fig. 7). The range of observed/predicted *k*_w_ ratios was 0.4 to 1.7, excluding the likely erroneous value for bentazon in Phase 2. Some uncertainty in the observed *k*_w_ values is associated with the 13 °C temperature decrease during all Phases, but *k*_w_ is expected to decrease by only a factor of 1.3 over this temperature range (Section S5 from Booij and Chen 2018). Although the average observed *k*_w_ agrees well with the Glanzmann et al. prediction, present data give no reason to prefer their recommended proportionality constant of 0.52 over the theoretical value of 0.664. Experimentally determined *k*_w_ values from alabaster dissolution or dissipation of performance reference compounds from silicone samplers would be a valuable addition for future experiments (Booij et al. 2017; Glanzmann et al. 2022).

### Biofouling effects

**Fig. 8:**
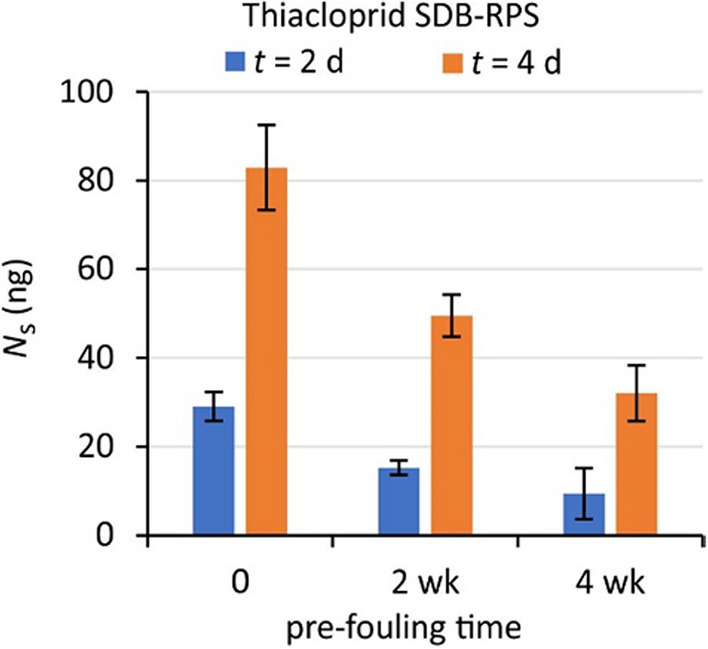
Effect of pre-fouling on accumulation of thiacloprid by SDB-RPS samplers in Channel A at *t* = 2 and 4 days, after pre-fouling for 0, 2, 4 weeks

**Fig. 9:**
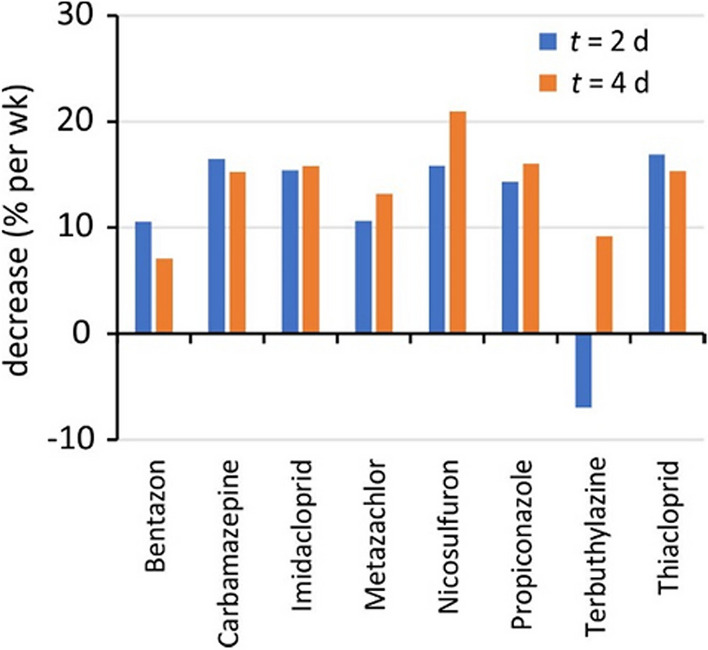
Reduction of accumulation by SDB-RPS samplers in Channel A at *t* = 2 and 4 days, after pre-fouling in Channel C

Allowing biofouling to develop on SDB-RPS samplers in Channel C, prior to exposure in Channel A, caused an appreciable reduction of accumulated amounts (Fig. 8, Figure S12-1). This reduction was 13 ± 6% per week of pre-fouling (relative to the amount for samplers without pre-fouling, Fig. 9). The biofouling effect for terbuthylazine is less pronounced than for other compounds, because of the non-zero background concentrations in Channel C for this compound.

The relatively compound-independent effect of biofouling is in line with an earlier suggestion that the fouling layer can be modelled as an immobilized water layer (Eq. 16). A crude estimate of the effective thickness of this layer can be obtained as follows. The overall mass transfer coefficient for WBL and sorbent is *k*_o_ = *R*_s_/*A*, which is for Phase 3 equal to 2.1 ± 0.8 L/(dm^2^ day) = 2.4 ± 1.0 µm/s (based on *R*_s_/*A* values for all compounds, Table 4). Four weeks of biofouling caused a reduction of approximately 50%, indicating that the biofouling resistance (*θ *^2^*d*_b_/[*φ* _b_ *D*_w_]) is equal to the combined resistances of WBL and sorbent. Adopting *D*_w_ = 350 µm^2^/s for 11 °C during Phase 3 yields an effective thickness of the fouling layer of *θ *_b_^2^*d*_b_/*φ* _b_ = 350/2.4 = 150 µm, which looks like a reasonable estimate in view of the low degree of visible fouling (Figure S12-2).

## Implications

The analytical sampling rate model was adequate for evaluating passive sampler responses to time-variable *C*_w_ of the studied compounds. The best performance was observed for SDB-RPS disks with a PES membrane, despite the occurrence of analyte sorption to PES. Sampling rates spanned a relatively narrow range for all analytes, lag times were minor, and TIWs were highest. This conclusion is provisional because this sampler type was only evaluated in Phase 3 of the present study. Additional study with sampler exposures to multiple peak concentration events is needed for this sampler design. The time-integrative capability of SDB-RPS samplers without a PES membrane is fair, but *R*_s_ is more strongly compound dependent and harder to understand. Silicone samplers are of little use for the time-integrative sampling of the present compounds because of their short TIWs.

The diffusion model for exposures to time-variable *C*_w_ may be useful when a more fundamental understanding of sampler-water exchange mechanisms is needed. Knowledge of these mechanisms allows to assess the effect of flow and temperature on the exchange kinetics, using models for the temperature and flow dependency of *k*_w_, and the temperature dependency of *K*’_sw_ and *D*_s_. In the present study, the model parameters (*k*_w_ and *K*’_sw_) were obtained by curve fitting of uptake and dissipation data. It would be interesting to test if calibration cost can be reduced by obtaining *K*’_sw_ from batch sorption experiments and calculating *k*_w_ from existing models.

The diffusion model is of limited use for practical application in routine monitoring. The better accuracy of this model only takes effect when exposure concentrations are either constant, or when time and duration of peak concentration events are known. This is usually not the case, but the diffusion model may be used to quantify the uncertainties that are associated with intermittent ambient concentrations, for example by comparing scenarios where peak concentrations occur at the beginning or at the end of the sampler exposure. The diffusion model may similarly be used to calculate the distribution of sampling rates that may be encountered, given the uncertainties in the occurrence of peak events and uncertainties in flow velocity and temperature at the exposure sites.

More importantly, the diffusion model may be used to identify sampler designs for which the transport resistance of the sorbent is much smaller than the combined resistance of membrane and WBL. In that case the numerical accuracy of the *R*_s_ model is the same as for the diffusion model, which greatly reduces the computational burden.

## Supplementary Information

Below is the link to the electronic supplementary material.

Supplementary file1 (DOCX 5437 KB)

## Data Availability

The datasets used and/or analyzed during the current study are available from the corresponding author on request.
