# Supplementary material for: Modelling passive sampling of hydrophilic compounds under time-variable aqueous concentrations
Source: Environ Sci Pollut Res Int. 2024 Aug 12;31(39):51844–57. doi: 10.1007/s11356-024-34460-x (PMC11374861; doi:10.1007/s11356-024-34460-x)
Supplement: Supplementary file 1 — Supplementary file1 (DOCX 5437 KB) [file 11356_2024_34460_MOESM1_ESM.docx]

**Supplementary information**

***Article:***

*Modelling passive sampling of hydrophilic compounds under time-variable aqueous concentrations*

***Journal:***

*Environmental Science and Pollution Research*

***Authors:***

*Benjamin Becker*, Christian Kochleus, Denise Spira, Julia Bachtin, Fabian König, Stefan Meinecke, Christel Möhlenkamp, Kees Booij*

***Corresponding author (*):***

*Benajmin.becker@bafg.de*

*Federal Institute of Hydrology (BfG), Am Mainzer Tor 1, 56068 Koblenz, Germany*

Table of Contents

[S1. Channels and exposure devices 2](#_Toc168143458)

[S2. Instrumental analysis and limits of detection/quantitation 4](#_Toc168143459)

[S3. Sampling rate model for time-dependent *C*_w_ 7](#_Toc168143460)

[S4. Diffusion model for time-dependent *C*_w_ 8](#_Toc168143461)

[S5. Considerations for parameter estimation by NLS 12](#_Toc168143462)

[S6. *C*_w_ modelling 13](#_Toc168143463)

[S7. Model plots for sampling with silicone 19](#_Toc168143464)

[S8. Flow effects for silicone samplers 20](#_Toc168143465)

[S9. Model plots for sampling by SDB-RPS samplers without membrane 21](#_Toc168143466)

[S10. Flow effects for SBD-RPS samplers 25](#_Toc168143467)

[S11. Membrane effects for SDB-RPS samplers 26](#_Toc168143468)

[S12. Biofouling effects for SDB-RPS samplers 29](#_Toc168143469)

[S13. References 30](#_Toc168143470)

# Channels and exposure devices


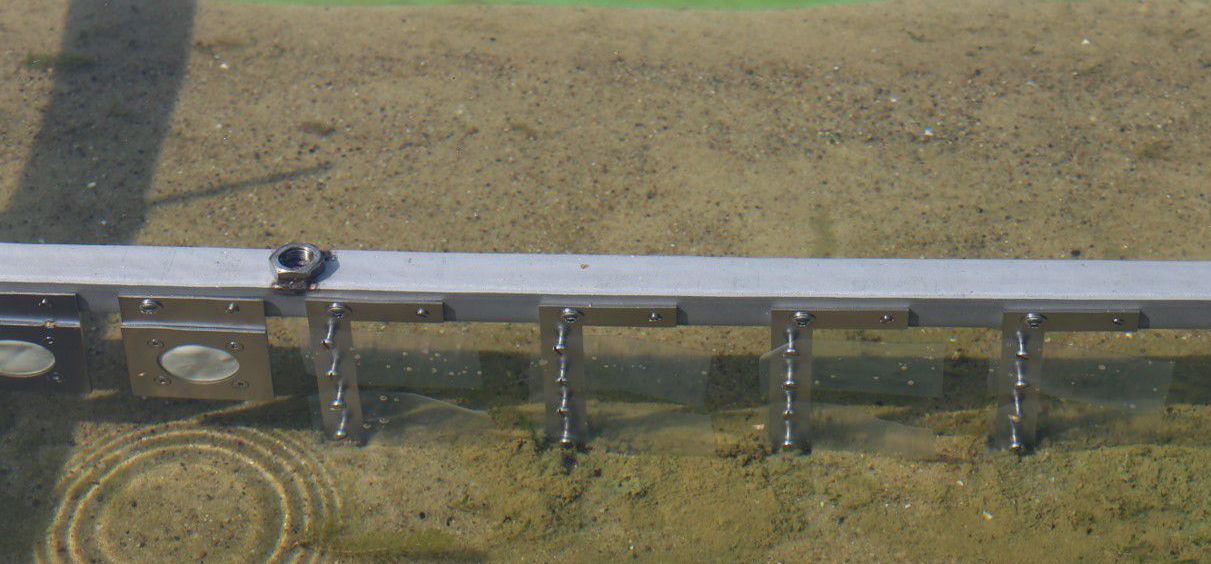


Figure S1‑1. Mounting of SDB-RPS samplers (left) and silicone (right).


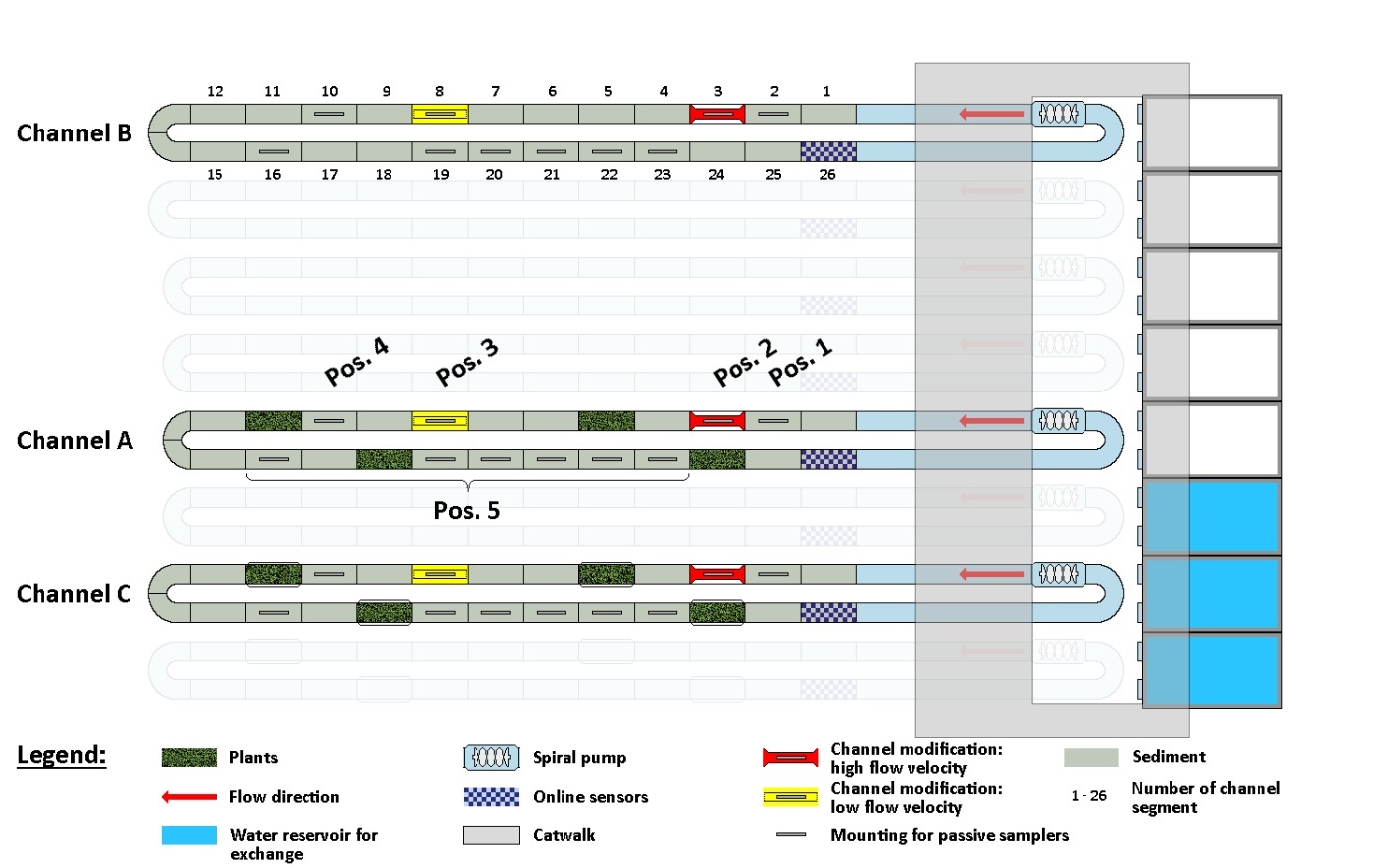


Figure S1‑2. Configuration of the exposure channels. (Schematic based on a template from UBA)


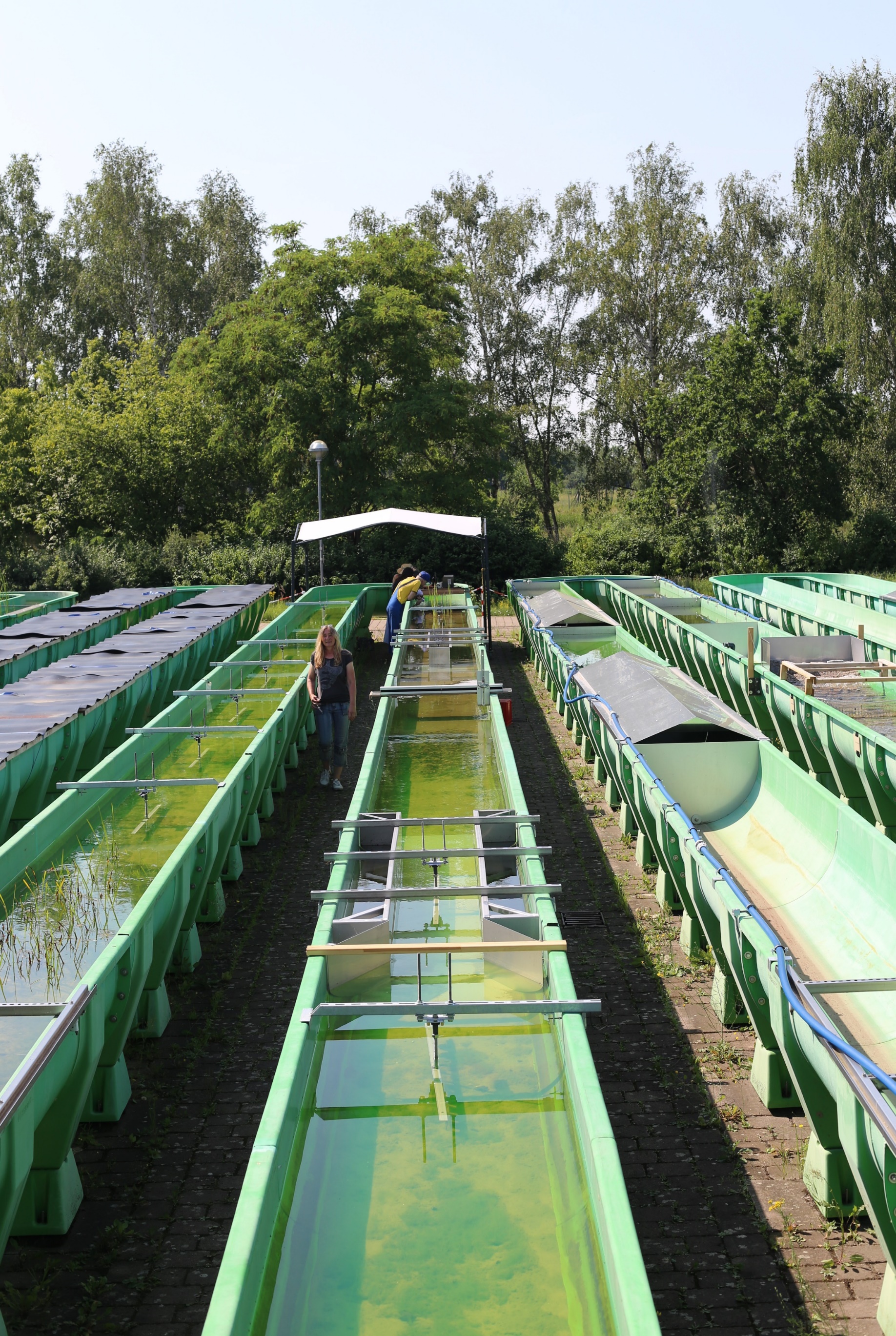

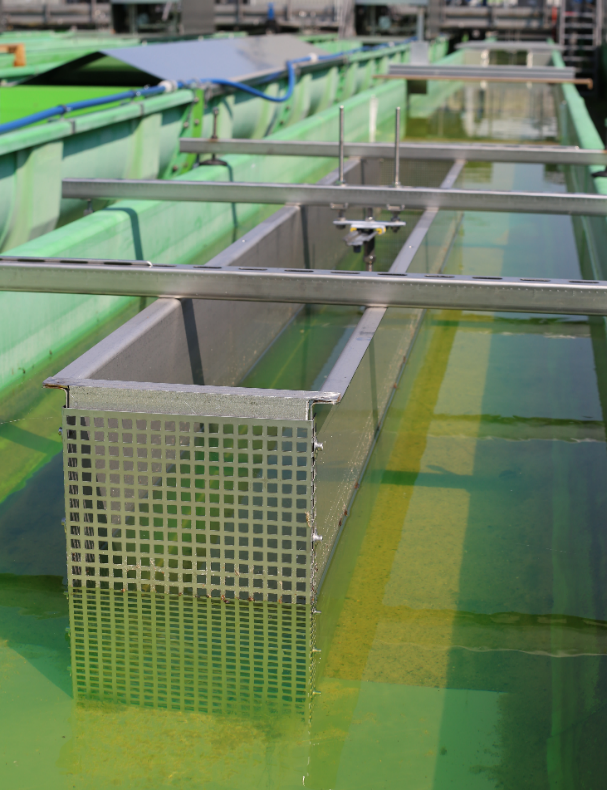


Figure S1‑3. Channel modification for generating flows of 18 cm/s (left) and 1 cm/s (right).

# **Instrumental analysis and limits of detection/quantitation**

The injection volume for analysis LC-MS/MS was 40 µL for sampler extracts, and 100 µL for water samples. The following gradient was run with a flow rate of 400 µL/min and eluents consisting of water with 0.4 mM ammonium acetate (A) and methanol (B): start with 10 % B for 0.5 minutes, increase to 95 % B in 3.5 minutes, hold for 9.5 minutes. In 0.2 minutes reduce to 10 % B and hold for 6.2 minutes.

Table 2-1 shows detector settings, target ions, and deuterated or C13-labeled internal standards for all analytes. For UV 326 no internal standard was used. Table 2-2 shows the LOD and LOQ of each analyte for the passive sampling extracts as well as for the water samples.

For the calibration standards 0.005, 0.01, 0.02, 0.05, 0.1, 0.25, 0.5, 1.0, 2.5, 5.0, 7.5, 10 ng mL^-1^ were used. Depending on the target concentration of the analyte the calibration curve was adjusted to the appropriate concentration range. Five µl, of a 200 ng mL^-1^ standard solution, was added to each sample, resulting in a concentration of 1 ng mL^-1^ in the extract.

At least two samples per sequence were spiked with a native standard solution (resulting in 0.5 ng mL^-1^ in extracts of silicone sheets and 1 ng mL^-1^ in extracts of SDB-RPS discs). The recovery was calculated from the difference of sample and spiked sample. Recoveries were within a range of 80 to 120 %.

Table 2‑1. Detector settings for HPLC-MS/MS analysis in ESI positive and ESI negative mode. Given are two ion transitions for each analyte, declustering potential (DP), entrance potential (EP), collision energy (CE) and cell exit potential (CXP).

| **ESI pos.** | **Analyte** | ***Q1 Mass (Da)*** | ***Q2 Mass (Da)*** | ***ID*** | ***DP*** | ***EP*** | ***CE*** | ***CXP*** |
| --- | --- | --- | --- | --- | --- | --- | --- | --- |
|  | Carbamazepine | 236.9 | 194.1 | Carbamazepine_1 | 81 | 10 | 25 | 8 |
|  |  | 236.9 | 192.1 | Carbamazepine_2 | 81 | 10 | 29 | 12 |
|  | Carbamazepine-13C15N | 238.9 | 194.0 | Carbamazepine_13C15N_1 | 66 | 10 | 25 | 20 |
|  |  | 238.9 | 192.0 | Carbamazepine_13C15N_2 | 66 | 10 | 29 | 12 |
|  | Flufenacet | 364.0 | 194.0 | Flufenacet _1 | 51 | 10 | 15 | 6 |
|  |  | 364.0 | 152.1 | Flufenacet_ 2 | 51 | 10 | 25 | 10 |
|  | Flufenacet-d4 | 368.0 | 198.0 | Flufenacet-d4_1 | 61 | 10 | 15 | 8 |
|  |  | 368.0 | 155.9 | Flufenacet-d4_2 | 61 | 10 | 23 | 10 |
|  | Imidacloprid | 256.0 | 175.1 | Imidacloprid_1 | 71 | 10 | 25 | 8 |
|  |  | 256.0 | 208.8 | Imidacloprid_2 | 71 | 10 | 19 | 18 |
|  | Imidacloprid-d4 | 260.0 | 179.1 | Imidacloprid-d4_1 | 71 | 10 | 25 | 8 |
|  |  | 260.0 | 212.8 | Imidacloprid-d4_2 | 71 | 10 | 19 | 18 |
|  | Metazachlor | 278.0 | 134.0 | Metazachlor_1 | 36 | 10 | 27 | 6 |
|  |  | 278.0 | 209.9 | Metazachlor_2 | 36 | 10 | 13 | 8 |
|  | Metazachlor-d6 | 284.0 | 140.2 | Metazachlor-d6_1 | 31 | 10 | 27 | 12 |
|  |  | 284.0 | 216.1 | Metazachlor-d6_2 | 31 | 10 | 15 | 16 |
|  | Nicosulfuron | 411.0 | 181.9 | Nicosulfuron_1 | 86 | 10 | 27 | 8 |
|  |  | 411.0 | 212.9 | Nicosulfuron_2 | 86 | 10 | 23 | 10 |
|  | Nicosulfuron-d6 | 417.1 | 218.8 | Nicosulfuron-d6_2 | 91 | 10 | 23 | 18 |
|  |  | 417.1 | 181.8 | Nicosulfuron-d6_1 | 91 | 10 | 25 | 12 |
|  | Pendimethalin | 282.1 | 211.9 | Pendimethalin_1 | 36 | 10 | 13 | 8 |
|  |  | 282.1 | 193.9 | Pendimethalin_2 | 36 | 10 | 23 | 8 |
|  | Pendimethalin-d5 | 287.0 | 213.2 | Pendimethalin-d5_1 | 16 | 10 | 15 | 14 |
|  |  | 287.0 | 194.1 | Pendimethalin-d5_2 | 16 | 10 | 25 | 10 |
|  | Propiconazole | 342.0 | 158.8 | Propiconazole_1 | 86 | 10 | 33 | 10 |
|  |  | 342.0 | 122.8 | Propiconazole_2 | 86 | 10 | 81 | 10 |
|  | Propiconazole-d3 | 345.0 | 162.0 | Propiconazole-d3_1 | 91 | 10 | 33 | 12 |
|  |  | 347.0 | 164.0 | Propiconazole-d3_2 | 91 | 10 | 23 | 20 |
|  | Terbuthylazine | 230.0 | 173.9 | Terbuthylazine_1 | 81 | 10 | 21 | 12 |
|  |  | 230.0 | 104.0 | Terbuthylazine_2 | 81 | 10 | 31 | 7 |
|  | Terbuthylazine-d5 | 235.0 | 179.0 | Terbuthylazine-d5_1 | 76 | 10 | 21 | 2 |
|  |  | 235.0 | 100.9 | Terbuthylazine-d5_2 | 76 | 10 | 33 | 26 |
|  | Thiacloprid | 252.9 | 125.9 | Thiacloprid_1 | 86 | 10 | 25 | 5 |
|  |  | 252.9 | 90.0 | Thiacloprid_2 | 86 | 10 | 53 | 8 |
|  | Thiacloprid-d4 | 256.9 | 126.0 | Thiacloprid-d4_1 | 56 | 10 | 27 | 4 |
|  |  | 256.9 | 89.9 | Thiacloprid-d4_2 | 56 | 10 | 45 | 10 |
|  | UV 326 | 316.1 | 260.1 | UV326_1 | 50 | 10 | 25 | 13 |
|  |  | 316.1 | 107.0 | UV326_2 | 130 | 10 | 44 | 7 |
|  |  |  |  |  |  |  |  |  |
| **ESI neg.** | **Analyte** | ***Q1 Mass (Da)*** | ***Q2 Mass (Da)*** | ***ID*** | ***DP*** | ***EP*** | ***CE*** | ***CXP*** |
|  | Bentazon | 238.8 | 131.9 | Bentazon_1 | -70 | -10 | -34 | -9 |
|  |  | 238.8 | 196.8 | Bentazon_2 | -70 | -10 | -28 | -15 |
|  | Bentazon-d7 | 245.9 | 132.0 | Bentazon-d7_1 | -65 | -10 | -32 | -7 |
|  |  | 245.9 | 182.0 | Bentazon-d7_2 | -65 | -10 | -26 | -17 |
|  | Diclofenac | 293.8 | 213.9 | Diclofenac_1 | -10 | -10 | -16 | -17 |
|  |  | 293.8 | 249.9 | Diclofenac_2 | -10 | -10 | -26 | -9 |
|  | Diclofenac-d4 | 297.8 | 254.0 | Diclofenac-d4_1 | -25 | -10 | -16 | -9 |
|  |  | 297.8 | 216.9 | Diclofenac-d4_2 | -25 | -10 | -28 | -15 |

Table 2‑2 Limits of detection (LOD) and limits of quantification (LOQ) in for passive samplers and water samples.

| Analyte | Sampler analysis  LOD [ng disk^-1^] | Sampler analysis  LOQ [ng disk^-1^] | Water analysis  LOD [ng mL^-1^] | Water analysis  LOQ [ng mL^-1^] |
| --- | --- | --- | --- | --- |
| Bentazon | 0.4 | 2.0 | 0.015 | 0.05 |
| Carbamazepine | 0.1 | 0.8 | 0.010 | 0.02 |
| Diclofenac | 0.8 | 8.0 | 0.005 | 0.20 |
| Flufenacet | 0.1 | 0.4 | 0.003 | 0.01 |
| Imidacloprid | 1.2 | 4.0 | 0.030 | 0.10 |
| Metazachlor | 0.1 | 0.4 | 0.015 | 0.05 |
| Nicosulfuron | 1.2 | 4.0 | 0.003 | 0.01 |
| Pendimethalin | 1.2 | 4.0 | 0.015 | 0.05 |
| Propiconazole | 0.1 | 0.4 | 0.003 | 0.01 |
| Terbuthylazine | 0.1 | 0.4 | 0.003 | 0.01 |
| Thiacloprid | 0.1 | 0.4 | 0.003 | 0.01 |
| UV 326 | 2.0 | 10 | 0.100 | 0.50 |

# Sampling rate model for time-dependent *C*_w_

With the sampling rate model it is assumed that the sampler-water flux is proportional to the difference in effective concentration (*C*_w_ - *C*_s_/*K*_sw_).

$\frac{dN_{\text{s}}}{dt}=R_{\text{s}}\left(C_{\text{w}}-\frac{C_{\text{s}}}{K_{\text{sw}}}\right)$ (

1

)

where *N*_s_ is the amount in the sampler, *C*_s_ is the concentration in the sampler, *R*_s_ is the sampling rate, and *K*_sw_ is the sampler-water partition coefficient in volume per mass units.

Substituting *N*_s_ = *m* *C*_s_ (where *m* is the sampler mass), rearranging, and writing *k*_e_=*R*_s_/(*mK*_sw_) to simplify notation gives

$\frac{dC_{\text{s}}}{dt}+k_{\text{e}}C_{\text{s}}=k_{\text{e}}K_{\text{sw}}C_{\text{w}}$ (

2

)

Eq. 2 has a particular solution (*y*_p_) and a complementary solution (*y*_c_), the full solution being *C*_s_ = *y*_c_ + *y*_p_. The complementary solution is obtained by setting the righthand side of eq. 2 to zero.

$\frac{dy_{\text{c}}}{dt}+k_{\text{e}}y_{\text{c}}=0$ (

3

)

$\frac{dy_{\text{p}}}{dt}+k_{\text{e}}y_{\text{p}}=k_{\text{e}}K_{\text{sw}}C_{\text{w}}$ (

4

)

The solution for *y*_c_ is

$y_{\text{c}}=a\exp\left(-k_{\text{e}}t\right)$ (

5

)

where *a* is an integration constant. The particular solution is obtained using the method of variation of parameters, which postulates that a solution can be found by writing *y*_p_ as the product of *y*_c_ and some function *f*(*t*). Substituting *y*_p_ = *y*_c_ *f*(*t*) in eq. 4 and rearranging gives

$f(t)\left[y'_{\text{c}}+k_{\text{e}}y_{\text{c}}\right]+y_{\text{c}}f'(t)=k_{\text{e}}K_{\text{sw}}C_{\text{w}}$ (

6

)

The term between brackets equals zero because of eq. 3, and *f*(*t*) is found by integration of *f* ′(t)

$f(t)=k_{\text{e}}K_{\text{sw}}\int_{0}^{t} \frac{C_{\text{w}}}{y_{\text{c}}} dt'$ (

7

)

The full solution *C*_s_ = *y*_c_+*y*_c_ *f*(*t*) is given by

$C_{\text{s}}(t)=\exp\left(-k_{\text{e}}t\right)\left(C_{\text{s0}}+ k_{\text{e}}K_{\text{sw}}\int_{0}^{t} \exp\left(k_{\text{e}}t'\right)C_{\text{w}}(t')dt'\right)$ (

8

)

where the integration constant *a* was obtained from the initial condition *C*_s_(0) = *C*_s0_. The integral can be evaluated analytically when *C*_w_ is a simple function of time, for example a polynomial or an exponential. Numerical integration can also be an option.

In present study, *C*_w_ in Channel A was modelled using

$C_{\text{w}}=\left(C_{\text{w0}}-C_{\text{w}\infty}\right)\exp\left(-\alpha t\right)+C_{\text{w}\infty}$ (

9

)

Substituting this model in eq. 8, integrating, and rearranging gives

$C_{\text{s}}(t)=K_{\text{sw}}C_{\text{w}\infty}+\left(C_{\text{s0}}-K_{\text{sw}}C_{\text{w}\infty}\right)\exp\left(-k_{\text{e}}t\right)+k_{\text{e}}K_{\text{sw}}\left(C_{\text{w0}}-C_{\text{w}\infty}\right)\frac{\exp\left(-\alpha t\right)-\exp\left(-k_{\text{e}}t\right)}{k_{\text{e}}-\alpha}$ (

10

)

For constant *C*_w_ scenarios the rightmost term vanishes, as *C*_w0_ = *C*_w∞_ in that case.

# Diffusion model for time-dependent *C*_w_

A diffusion model for uptake in a sorbent under time-variable concentrations in water and non-uniform initial distributions in the sorbent is given by Mikhailov and Özişik (1984, section 7.2) for planar, cylindrical and spherical configurations. The derivation for sheets is summarized here for the convenience of the reader.

Assuming that Fick’s second law applies, transport in the sorbent is governed by the differential equation (DE)

DE: $\frac{\partial C_{\text{s}}\left(x,t\right)}{\partial t}=D_{\text{s}}\frac{\partial^{2}C_{\text{s}}\left(x,t\right)}{\partial x^{2}}$ (

11

)

where *D*_s_ is the diffusion coefficient in the sorbent, and *C*_s_ is given in amount per unit volume of sorbent.

The first boundary condition (BC1) prescribes that the concentration gradient equals zero at the impermeable wall of the SDB-RPS housing or the center of the silicone sheets.

BC1: $\left.\frac{\partial C_{\text{s}}\left(x,t\right)}{\partial x}\right|_{x=0}=0$ (

12

)

The second boundary condition (BC2) prescribes that the flux out of the water is equal to the flux into the sorbent

BC2: $\left.D_{\text{s}}\frac{\partial C_{\text{s}}\left(x,t\right)}{\partial x}\right|_{x=L}=k_{\text{w}}\left(C_{\text{w}}\left(t\right)-\frac{C_{\text{s}}(L,t)}{K_{\text{sw}}^{'}}\right)$ (

13

)

where *K*’_sw_ is the sorption coefficient in volume per volume units, *C*_w_ is some function of time, and *L* is the thickness of one-sided exposed samplers or the half-thickness for two-sided exposed samplers.

The initial condition (IC) is that the concentration in the sorbent is some function of space.

IC: $C_{\text{s}}\left(x,0\right)=F(x)$ (

14

)

Dimensionless time (*τ*) and space (*X*) are useful to simplify notation

$X=\frac{x}{L}$ (

15

)

$\tau=\frac{D_{\text{s}}t}{L^{2}}$ (

16

)

Substituting in equations 11 through 14 gives

DE: $\frac{\partial C_{\text{s}}\left(X,\tau\right)}{\partial\tau}=\frac{\partial^{2}C_{\text{s}}\left(X,\tau\right)}{\partial X^{2}}$ (

17

)

BC1: $\left.\frac{\partial C_{\text{s}}\left(X,\tau\right)}{\partial X}\right|_{X=0}=0$ (

18

)

BC2: $\left.BiC_{\text{s}}\left(1,\tau\right)+\frac{\partial C_{\text{s}}\left(X,\tau\right)}{\partial X}\right|_{X=1}=BiK_{\text{sw}}^{'}C_{\text{w}}\left(\tau\right)$ (

19

)

where Bi is the Biot number *k*_w_*L*/(*K*’_sw_ *D*_s_).

IC: $C_{\text{s}}\left(X,0\right)=F(X)$ (

20

)

Mikhailov and Özişik assume that *C*_s_(*X*,*τ*) can be written as

$C_{\text{s}}\left(X,\tau\right)=\sum_{n=1}^{\infty} \Phi_{\text{n}}\left(\tau\right) \Psi_{\text{n}}\left(X\right)$ (

21

)

The justification for this assumption is that it turns out to work.

The functions Ψ_n_(X) are solutions of the boundary value problem

DE: $\frac{d^{2}\Psi\left(X\right)}{dX^{2}}=-\mu^{2}\Psi\left(X\right)$ (

22

)

BC1: $\left.\frac{d\Psi\left(X\right)}{dX}\right|_{X=0}=0$ (

23

)

BC2: $Bi \Psi\left(1\right)+\left.\frac{d\Psi\left(X\right)}{dX}\right|_{X=1}=0$ (

24

)

Eqs. 22-24 are a Sturm-Liouville boundary value problem, with has a series of eigenfunctions Ψ_n_(X) as a solution. The eigenfunctions have the property that

$\int_{0}^{1} \Psi_{\text{n}}\left(X\right)\Psi_{\text{m}}\left(X\right)dX=\left\{\begin{matrix} 0 \text{if} \text{m}\neq\text{n} \\ F_{\text{n}} \text{if} \text{m}=\text{n} \end{matrix}\right.$ (

25

)

where *F*_n_^½^ is called the norm of Ψ_n_(*X*). The proof of eq. 25 can be found throughout on the internet, or in Mikhailov and Özişik (1984, section 2.1).

The solution of Φ_n_(*τ*) is evaluated first (the solution of the Ψ_n_(*X*) is given below). Multiplying eq. 21 by Ψ_m_(*X*), and integrating over space gives

$\int_{0}^{1} \Psi_{\text{m}}\left(X\right) C_{\text{s}}\left(X,\tau\right)dX=\sum_{n=1}^{\infty} \Phi_{\text{n}}\left(\tau\right) \int_{0}^{1} \Psi_{\text{m}}\left(X\right)\Psi_{\text{n}}\left(X\right)dX$ (

26

)

The integral on the right equals zero for all n ≠ m because of eq. 25, and the functions Φ_n_(*τ*) are therefore given by

$\Phi_{\text{n}}\left(\tau\right)=\frac{\int_{0}^{1} \Psi_{\text{n}}\left(X\right) C_{\text{s}}\left(X,\tau\right)dX}{\int_{0}^{1} \Psi_{\text{n}}\left(X\right)\Psi_{\text{n}}\left(X\right)dX}=\frac{\tilde{C}_{\text{s,n}}\left(\tau\right)}{F_{\text{n}}}$ (

27

)

where $\tilde{C}_{\text{s,n}}\left(\tau\right)$is the finite integral transform of concentrations in the sorbent.

$\tilde{C}_{\text{s,n}}\left(\tau\right)=\int_{0}^{1} \Psi_{\text{n}}\left(X\right) C_{\text{s}}\left(X,\tau\right)dX$ (

28

)

Substituting eq. 27 in eq. 21 gives an expression for the local concentrations in the sorbent.

$C_{\text{s}}\left(X,\tau\right)=\sum_{n=1}^{\infty} \frac{1}{F_{\text{n}}}\tilde{C}_{\text{s,n}}\left(\tau\right) \Psi_{\text{n}}\left(X\right)$ (

29

)

The integral transform (eq. 28) is used to convert the partial differential equation 17 in to an ordinary differential equation that is more easily solved. Equation 17 is multiplied by Ψ_n_(*X*) and integrated over space

$\frac{d\tilde{C}_{\text{s,n}}\left(\tau\right)}{d\tau}=\int_{0}^{1} \Psi_{\text{n}}\left(X\right)\frac{\partial^{2}C_{\text{s}}\left(X,\tau\right)}{\partial X^{2}}dX$ (

30

)

Similarly, eq. 22 is multiplied by *C*_s_(*X*,*τ*) and integrated over space

$\mu_{\text{n}}^{2} \tilde{C}_{\text{s,n}}\left(\tau\right)=-\int_{0}^{1} C_{\text{s}}\left(X,\tau\right)\frac{d^{2}\Psi_{\text{n}}\left(X\right)}{dX^{2}}dX$ (

31

)

The righthand side of eq. 30 is integrated by parts.

$\frac{d\tilde{C}_{\text{s,n}}\left(\tau\right)}{d\tau} =\left[\Psi_{\text{n}}\left(X\right)\frac{\partial C_{\text{s}}\left(X,\tau\right)}{\partial X}\right]_{0}^{1}-\int_{0}^{1} \frac{d\Psi_{\text{n}}\left(X\right)}{dX} \frac{\partial C_{\text{s}}\left(X,\tau\right)}{\partial X}dX$ (

32

)

The righthand side of eq. 31 is also integrated by parts

$\mu_{\text{n}}^{2} \tilde{C}_{\text{s,n}}\left(\tau\right)=-\left[C_{\text{s}}\left(X,\tau\right)\frac{d\Psi_{\text{n}}\left(X\right)}{dX}\right]_{0}^{1}+\int_{0}^{1} \frac{d\Psi_{\text{n}}\left(X\right)}{dX}\frac{\partial C_{\text{s}}\left(X,\tau\right)}{\partial X}dX$ (

33

)

The integrals cancel after adding eqs. 32 and 33.

$\frac{d\tilde{C}_{\text{s,n}}\left(\tau\right)}{d\tau}+\mu_{\text{n}}^{2} \tilde{C}_{\text{s}}\left(\tau\right)=\left[\Psi_{\text{n}}\left(X\right)\frac{\partial C_{\text{s}}\left(X,\tau\right)}{\partial X}\right]_{0}^{1}-\left[C_{\text{s}}\left(X,\tau\right)\frac{d\Psi_{\text{n}}\left(X\right)}{dX}\right]_{0}^{1}$ (

34

)

The gradients at *X* = 0 and *X* = 1 are evaluated from the boundary conditions (eqs. 18, 19 ,23, and 24).

$\frac{d\tilde{C}_{\text{s,n}}\left(\tau\right)}{d\tau}+\mu_{\text{n}}^{2} \tilde{C}_{\text{s,n}}\left(\tau\right)=\Psi_{\text{n}}\left(1\right)Bi K_{\text{sw}}^{'}C_{\text{w}}\left(\tau\right)$ (

35

)

which can be solved using the method of variation of parameters.

$\tilde{C}_{\text{s,n}}\left(\tau\right)=\exp\left(-\mu_{\text{n}}^{2} \tau\right)\left[\tilde{C}_{\text{s,n}}\left(0\right)+\Psi_{\text{n}}\left(1\right)Bi K_{\text{sw}}^{'}\int_{0}^{\tau} C_{\text{w}}\left(\tau'\right)\exp\left(+\mu_{\text{n}}^{2} \tau'\right)d\tau'\right]$ (

36

)

where $\tilde{C}_{\text{s,n}}\left(0\right)$ is the integral transform of the initial concentration in the sorbent. Substituting eq. 36 in eq. 29 yields

$C_{\text{s}}\left(X,\tau\right)=\sum_{n=1}^{\infty} \frac{1}{F_{\text{n}}} \Psi_{\text{n}}\left(X\right)\exp\left(-\mu_{\text{n}}^{2} \tau\right)\left[\tilde{C}_{\text{s,n}}\left(0\right)+\Psi_{\text{n}}\left(1\right)Bi K_{\text{sw}}^{'}\int_{0}^{\tau} C_{\text{w}}\left(\tau'\right)\exp\left(\mu_{\text{n}}^{2} \tau'\right)d\tau'\right]$ (

37

)

Solutions of the differential equation (eq. 22) for Ψ_n_(*X*) that satisfy the boundary condition at *X* = 0 (eq. 23) are

$\Psi_{\text{n}}\left(X\right)=\cos\left(\mu_{\text{n}}X\right)$ (

38

)

The values of *μ*_n_ follow from the boundary condition at *X* = 1 (eq. 24)

$\cot\left(\mu_{\text{n}}\right)-\frac{\mu_{\text{n}}}{Bi}=0$ (

39

)

The *μ*_n_ have to be evaluated numerically, by the bisection method (Hahn and Özişik, 2012, section 2.4) or otherwise.

The full solution to eqs. 17-20 is

$C_{\text{s}}\left(X,\tau\right)=\sum_{n=1}^{\infty} \frac{1}{F_{\text{n}}}\cos\left(\mu_{\text{n}}X\right)e^{-\mu_{\text{n}}^{2} \tau}\left[\tilde{C}_{\text{s,n}}\left(0\right)+\mu_{\text{n}} \sin\left(\mu_{\text{n}}\right)K_{\text{sw}}^{'}\int_{0}^{\tau} C_{\text{w}}\left(\tau'\right)e^{\mu_{\text{n}}^{2} \tau'}d\tau'\right]$ (

40

)

$F_{\text{n}}=\int_{0}^{1} \cos^{2}\left(\mu_{\text{n}}X\right)dX=\frac{1}{2}+\frac{\cos\left(\mu_{\text{n}}\right)\sin\left(\mu_{\text{n}}\right)}{2\mu_{\text{n}}}$ (

41

)

$\tilde{C}_{\text{s,n}}\left(0\right)=\int_{0}^{1} \cos\left(\mu_{\text{n}}X\right)C_{\text{s}}\left(X,0\right) dX$ (

42

)

The space-averaged concentration *C*_s_(*τ*) in the sorbent is obtained by integrating eq. 40 over space.

$C_{\text{s}}\left(\tau\right)=\sum_{n=1}^{\infty} \frac{\sin\left(\mu_{\text{n}}\right)}{F_{\text{n}}\mu_{\text{n}}}e^{-\mu_{\text{n}}^{2} \tau}\left[\tilde{C}_{\text{s,n}}\left(0\right)+\mu_{\text{n}} \sin\left(\mu_{\text{n}}\right)K_{\text{sw}}^{'}\int_{0}^{\tau} C_{\text{w}}\left(\tau'\right)e^{\mu_{\text{n}}^{2} \tau'}d\tau'\right]$ (

43

)

Application of the diffusion model for subsequent stages requires evaluation of the integral transform of the initial concentration at each new stage i+1. This can be evaluated by taking the integral transform of eq. 40 at the final time *τ*_f,i_ of the previous stage i. The m^th^ term of the integral transform $\tilde{C}_{\text{s,m,i+1}}\left(0\right)$ equals

$\begin{matrix} \tilde{C}_{\text{s,m,i+1}}\left(0\right)=\tilde{C}_{\text{s,m,i}}\left(\tau_{\text{f,i}}\right)=\int_{0}^{1} C_{\text{s}}\left(X,\tau_{\text{f,i}}\right)\cos\left(\mu_{\text{m}}X\right)dX \\ =\sum_{n=1}^{\infty} \left\{\frac{1}{F_{\text{n}}}\int_{0}^{1} \cos\left(\mu_{\text{n}}X\right)\cos\left(\mu_{\text{m}}X\right)dX \right\}e^{-\mu_{\text{n}}^{2} \tau_{\text{f,i}}}\left[\tilde{C}_{\text{s,n,i}}\left(0\right)+\mu_{\text{n}} \sin\left(\mu_{\text{n}}\right)K_{\text{sw}}^{'}\int_{0}^{\tau_{\text{f,i}}} C_{\text{w}}\left(\tau'\right)e^{\mu_{\text{n}}^{2} \tau'}d\tau'\right] \end{matrix}$ (

44

)

The term between curly braces equals zero for n ≠ m and equals 1 for n = m, because of the orthogonality properties of the eigenfunctions (eq. 25). All terms in the summation therefore equal zero, except the term with n = m.

$\tilde{C}_{\text{s,m,i+1}}\left(0\right)=\int_{0}^{1} C_{\text{s}}\left(X,\tau_{\text{f,i}}\right)\cos\left(\mu_{\text{m}}X\right)dX =e^{-\mu_{\text{m}}^{2} \tau_{\text{f,i}}}\left[\tilde{C}_{\text{s,m,i}}\left(0\right)+\mu_{\text{m}} \sin\left(\mu_{\text{m}}\right)K_{\text{sw}}^{'}\int_{0}^{\tau_{\text{f,i}}} C_{\text{w}}\left(\tau'\right)e^{\mu_{\text{m}}^{2} \tau'}d\tau'\right]$ (

45

)

which is evaluated by multiplying the m^th^ term of *C*_s_(*τ*_f,i_) in eq. 43 by *F*_m_*μ*_m_/sin(*μ*_m_).

$\tilde{C}_{\text{s,m,i+1}}\left(0\right)=\frac{F_{\text{m}}\mu_{\text{m}}}{\sin\left(\mu_{\text{m}}\right)}C_{\text{s,m,i}}\left(\tau_{\text{f,i}}\right)$ (

46

)

This calculation scheme is only applicable if the eigenvalues *μ*_m_ are the same for each stage. This is the case for present study, because Biot numbers are the same in all stages.

The *C*_w_ model for present study was incorporated in the diffusion model as follows. Combining the *C*_w_ model (eq. 5 from the main text) with eq. 43 and evaluating the integral yields

$C_{\text{s}}\left(\tau\right)=\sum_{n=1}^{\infty} \frac{\sin\left(\mu_{\text{n}}\right)}{F_{\text{n}}\mu_{\text{n}}}e^{-\mu_{\text{n}}^{2} \tau}\left[\tilde{C}_{\text{s,n}}\left(0\right)+\mu_{\text{n}} \sin\left(\mu_{\text{n}}\right)K_{\text{sw}}^{'}\left\{\frac{\left(C_{\text{w0}}-C_{\text{w}\infty}\right)}{-\beta+\mu_{\text{n}}^{2}}\left(e^{\left(-\beta+\mu_{\text{n}}^{2} \right)\tau}-1\right)+\frac{C_{\text{w}\infty}}{\mu_{\text{n}}^{2}}\left(e^{\mu_{\text{n}}^{2} \tau}-1\right)\right\}\right]$ (

47

)

where *β* = *αL*^2^/*D*_s_.

For coding purposes it is better to bring the term exp(−*μ*_n_^2^*τ*) within the square brackets of eq.47, because numerical overflow occurs in exp(*x*) if *x* > 709.

$C_{\text{s}}\left(\tau\right)=\sum_{n=1}^{\infty} \frac{\sin\left(\mu_{\text{n}}\right)}{F_{\text{n}}\mu_{\text{n}}}\left[\tilde{C}_{\text{s,n}}\left(0\right)e^{-\mu_{\text{n}}^{2} \tau}+\mu_{\text{n}} \sin\left(\mu_{\text{n}}\right)K_{\text{sw}}^{'}\left\{\frac{\left(C_{\text{w0}}-C_{\text{w}\infty}\right)}{-\beta+\mu_{\text{n}}^{2}}\left(e^{-\beta\tau}-e^{-\mu_{\text{n}}^{2} \tau}\right)+\frac{C_{\text{w}\infty}}{\mu_{\text{n}}^{2}}\left(1-e^{-\mu_{\text{n}}^{2} \tau}\right)\right\}\right]$ (

48

)

Modelling of stages with constant *C*_w_ (e.g., the pre-spike stage) were dealt with by setting *C*_w0_ = *C*_w∞_, which causes the first term within curly braces at the right-hand side of eq. 48 to be zero.

# Considerations for parameter estimation by NLS

Finding the optimal model parameters with nonlinear least-squares estimation included multiple steps. Estimation was most laborious for application of the diffusion model to data for accumulation by silicone samplers, because three adjustable parameters had to be optimized (*k*_w_, *D*_s_, and *K*’_sw_). This often caused the parameter optimization to terminate prematurely. To better understand the error structure, *D*_s_ and *K*’_sw_ were optimized at fixed values of *k*_w_, and the residual sum of squares was plotted versus *k*_w_. This procedure was then repeated by optimizing *k*_w_ and *K*’_sw_ at fixed *D*_s_, and by optimizing *D*_s_ and *k*_w_ at fixed *K*’_sw_. An example is shown in Figure S5‑1 (top row) for carbamazepine uptake by silicone samplers. The SSQ shows a clear minimum when plotted versus *D*_s_ and *K*’_sw_, but attains a plateau value when plotted versus *k*_w_. This indicates that *k*_w_ is larger than approximately 5 µm/s and that its exact value cannot be determined. The physical interpretation is that the uptake was fully controlled by the sorbent at the present flow velocity.

For thiacloprid, the SSQ has a shallow minimum of 3583 at *k*_w_ = 0.082 µm/s and levels off at higher *k*_w_ (, bottom row). The partial *F*-test was used to evaluate if this minimum could have occurred by chance. Adopting a very high *k*_w_ of 1000 µm/s essentially transforms the mixed rate control model (3 adjustable parameters) to a sorbent-control model (2 adjustable parameters).

| model | parameters | SSQ | degrees of freedom |
| --- | --- | --- | --- |
| full model | *k*_w_, *D*_s_, and *K*’_sw_ | 3583 | 17 |
| reduced model | *D*_s_, and *K*’_sw_ | 3855 | 18 |

$$F=\frac{\frac{SSQ_{\text{reduced}}-SSQ_{\text{full}}}{df_{\text{reduced}}-df_{\text{full}}}}{\frac{SSQ_{\text{full}}}{df_{\text{full}}}}=\frac{\frac{3855-3583}{18-17}}{\frac{3583}{17}}=1.29$$

The right-tailed probability of *F* = 1.29 equals 0.13, which means that including *k*_w_ as an adjustable parameter does not yield a significantly better model fit.


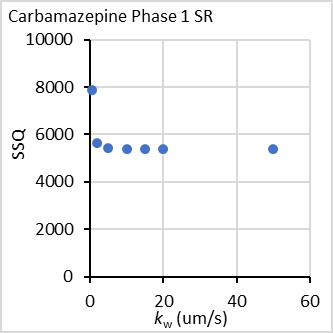

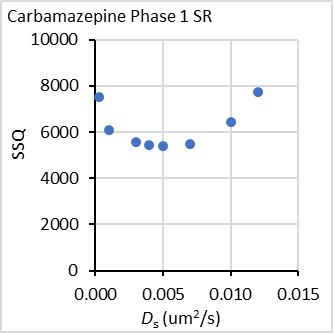

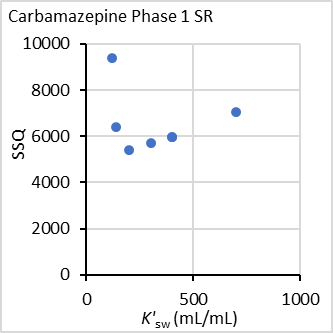


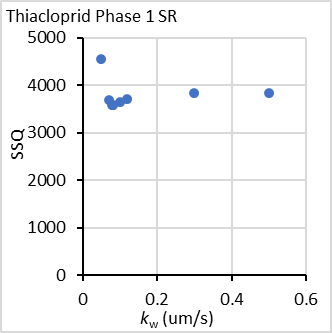

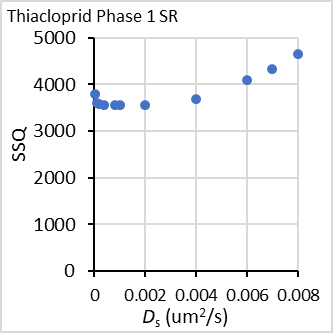

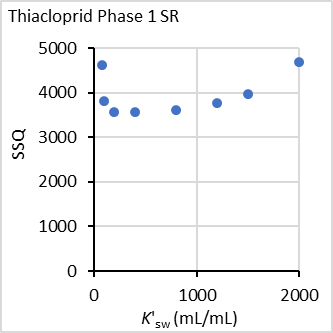


Figure S5‑1. Residual sum of squares (SSQ) with applying the diffusion model to silicone-water exchange of Carbamazepine (top) and Thiacloprid (bottom) in Phase 1, plotted versus *k*_w_, *D*_s_, and *K*’_sw_, with optimized values of the other parameters.

# *C*_w_ modelling

Aqueous concentrations (*C*_w_) in Channel A were modelled as

$C_{\text{w}}=\left(C_{\text{w0}}-C_{\text{w}\infty}\right)\exp\left(-\alpha t\right)+C_{\text{w}\infty}$ (

49

)

where *C*_w0_ is the concentration at the time of spiking, *C*_w∞_ is the limiting *C*_w_ at *t*→∞, and α is a first order decay constant.

The mass balance (*MB*) at the time of spiking was evaluated from

$MB=\frac{C_{\text{w0}}}{C_{\text{w,pre-spike}}+C_{\text{w0,spike}}}\times100\%$ (

50

)

where (*C*_w,pre-spike_) is the concentration immediately before spiking and *C*_w,0,spike_ is the calculated increase in *C*_w_ (spike amount divided by channel volume).

***C*_w_ modelling Phase 1**

**Channel A after spiking.** Modelling results for Channel A after spiking are summarized in and Figure S6‑1. *C*_w_ data for diclofenac, flufenacet, pendimethalin, propiconazole, and UV 326 were considered to be insufficiently reliable for the modelling of sampler-water exchange kinetics, because of poor mass balances at the time of spiking, or very fast disappearance (rows with red font in )

**Channel A before spiking.** *C*_w_ = 0 was adopted for the pre-spike period, except for terbuthylazine, for which measured *C*_w_ values > LOQ were observed.

**Channel B.** Constant background concentrations were measured for terbuthylazine in Channel B (0.12 ng/mL). Background concentrations of metazachlor in Channel B were set equal to the LOQ (0.005 ng/mL). The justification for this is that amounts in silicone samplers reached a plateau value of 7 ± 2 ng at *t*> 5 d, which is only possible when *C*_w_ > 0. (Section S7, metazachlor plots). For all other compounds a background *C*_w_ = 0 was adopted.

**Table S6‑1.** *C*_w_ modelling results for Phase 1. Compounds on rows with red font were excluded from further modelling because of poor mass balance (<80 %) or insufficient data. Δ*C*_w,spike_ = expected *C*_w_ increase at the time of spiking (spiked amount divided by channel volume). Other parameters are defined near eqs. 49 and 50 above.

|  | Δ*C*_w,spike_ | *C*_w,pre-spike_ | *C*_w0_ | *C*_w∞_ | α | Mass balance | n | s |
| --- | --- | --- | --- | --- | --- | --- | --- | --- |
|  | (ng/mL) | (ng/mL) | (ng/mL) | (ng/mL) | (d^−1^) | (%) |  | (ng/mL) |
| Bentazon | 0.5 | 0 | 0.47 | 0 | 0.096 | 93 | 65 | 0.03 |
| Carbamazepine | 1.0 | 0 | 0.82 | 0 | 0.017 | 82 | 65 | 0.06 |
| Diclofenac | 1.0 | 0 | 0.71 | 0 | 15 | 71 | 20 | 0.05 |
| Flufenacet | 0.5 | 0 | 0.34 | 0 | 0.040 | 67 | 65 | 0.05 |
| Imidacloprid | 0.5 | 0 | 0.41 | 0 | 0.64 | 82 | 56 | 0.04 |
| Metazachlor | 0.5 | 0 | 0.46 | 0 | 0.035 | 91 | 65 | 0.03 |
| Nicosulfuron | 0.5 | 0 | 0.52 | 0 | 0 | 103 | 65 | 0.06 |
| Pendimethalin | 0.5 | 0 | 0.05 | 0 | 0.66 | 10 | 36 | 0.01 |
| Propiconazole | 0.5 | 0 | 0.29 | 0 | 0.045 | 59 | 65 | 0.04 |
| Terbuthylazine | 0.5 | 0.12 | 0.51 | 0.12 | 0 | 82 | 65 | 0.04 |
| Thiacloprid | 0.5 | 0 | 0.40 | 0 | 0.028 | 80 | 65 | 0.03 |
| UV 326 | 0.5 | - | - | - | - | - | 4 |  |

***C*_w_ modelling Phase 2**

**Channel A after spiking.** For each compound, the *C*_w_ model (eq. 49) was fitted to the data using event-specific peak concentrations (*C*_w0,i_) and a common decay constant for all spike events as adjustable parameters. *C*_w∞_ was included as an adjustable parameter when measured *C*_w_ levelled off to a plateau value. This was observed for carbamazepine, metazachlor, terbuthylazine, and thiacloprid (). For other compounds *C*_w∞_ = 0 was adopted.

Results of the modelling are summarized in and . *C*_w_ data for diclofenac, flufenacet, pendimethalin, propiconazole, and UV 326 were considered to be insufficiently reliable for the modelling of sampler-water exchange kinetics, because of poor mass balances at the time of spiking, or very fast disappearance (rows with red font in ). *C*_w_ data for nicosulfuron were still used to allow comparison of sampler kinetics with results for Phases 1 and 3.

**Channel A before spike 1**. Background *C*_w_ > LOQ during the pre-spike period were observed for carbamazepine (0.031 ng/mL), terbuthylazine (0.046 ng/mL) and thiacloprid (0.0065 ng/mL). The pre-spike *C*_w_ of metazachlor was set equal to the LOQ (0.005 ng/mL), to account for the amounts in SDB-RPS samplers just before spiking (duplicate values of 2.0 ng at t = 0.98 d). For all other compounds *C*_w_ = 0 was adopted.

**Table S6‑2.** *C*_w_ modelling results for Phase 2. Compounds on rows with red font were excluded from further modelling because of poor average mass balance (<80 % or >120 %) or insufficient data. Δ*C*_w,spike_ = expected *C*_w_ increase at the time of spiking (spiked amount divided by channel volume). Other parameters are defined near eqs. 49 and 50 above.

|  | Δ*C*_w,spike_  Peak 1&2)^1^ | Peak 1 *C*_w0,1_ | Peak 2 *C*_w0,2_ | Peak 3 *C*_w0,3_ | C_w∞_ | α | Peak 1  Mass  balance | Peak 2  Mass  balance | Peak 3  Mass  balance | n | s |
| --- | --- | --- | --- | --- | --- | --- | --- | --- | --- | --- | --- |
|  | (ng/mL) | (ng/mL) | (ng/mL) | (ng/mL) | (ng/mL) | (d^−1^) | (%) | (%) | (%) |  | (ng/mL) |
| Bentazon | 0.05 | 0.061 | 0.059 | 0.099 | 0 | 0.975 | 121 | 100 | 96 | 7 | 0.003 |
| Carbamazepine | 0.05 | 0.077 | 0.062 | 0.109 | 0.011 | 2.28 | 96 | 100 | 98 | 32 | 0.004 |
| Diclofenac | 0.10 | 0.136 | 0.139 | 0.193 | 0 | 4.65 | 136 | 139 | 97 | 5 | 0.004 |
| Flufenacet | 0.05 | 0.043 | 0.019 | 0.049 | 0 | 1.80 | 86 | 37 | 49 | 13 | 0.005 |
| Imidacloprid | 0.05 | 0.047 | 0.067 | 0.098 | 0 | 2.09 | 94 | 132 | 97 | 18 | 0.007 |
| Metazachlor | 0.05 | 0.053 | 0.058 | 0.108 | 0.007 | 1.92 | 105 | 100 | 101 | 32 | 0.004 |
| Nicosulfuron | 0.05 | 0.080 | 0.078 | 0.122 | 0 | 1.67 | 160 | 148 | 121 | 9 | 0.014 |
| Pendimethalin | 0.05 | no data | - | - | - | - | - | - | - | - | - |
| Propiconazole | 0.05 | 0.045 | 0.032 | 0.047 | 0 | 0.25 | 91 | 41 | 41 | 10 | 0.007 |
| Terbuthylazine | 0.05 | 0.100 | 0.057 | 0.103 | 0.014 | 2.01 | 104 | 87 | 90 | 32 | 0.005 |
| Thiacloprid | 0.05 | 0.053 | 0.056 | 0.099 | 0.008 | 1.95 | 94 | 95 | 92 | 29 | 0.003 |
| UV 326 | 0.10 | no data | - | - | - | - | - | - | - | - | - |

)^1^ Twice as high for Peak 3

***C*_w_ modelling phase 3**

**Channel A after spiking.** Modelling results for Channel A after spiking are summarized in and . *C*_w_ data for flufenacet, pendimethalin, propiconazole, and UV 326 were considered to be insufficiently reliable, because of poor mass balances at the time of spiking (rows with red font in ). The mass balance of terbuthylazine spiking was unsatisfactory, but this compound was still included for consistency with results for Phases 1 and 2.

**Channel A before spiking.** *C*_w_ values > LOQ were observed for carbamazepine and terbuthylazine in the pre-spike period. Nonzero *C*_w_ were also adopted for metazachlor (0.005 ng/mL), nicosulfuron (0.005 ng/mL), and thiacloprid (0.003 ng/mL), to match the amounts in SDB-RPS samplers at the time of spiking. *C*_w_ = 0 was adopted for the remaining compounds.

**Channel B.** Concentrations of terbuthylazine were 0.010 ng/mL. For metazachlor a value equal to the LOQ of 0.005 ng/mL was adopted, because no dissipation was observed for this compound in Channel B. *C*_w_ = 0 was adopted for all other compounds.

**Table S6‑3.** *C*_w_ modelling results for Phase 3. Compounds on rows with red font were excluded from further modelling because of poor mass balance (<80 % or >120 %) or insufficient data. Δ*C*_w,spike_ = expected *C*_w_ increase at the time of spiking (spiked amount divided by channel volume). Other parameters are defined near eqs. 49 and 50 above.

|  | *C*_w,spike_ | *C*_w,pre-spike_ | *C*_w0_ | *C*_w∞_ | α | mass balance | n | s |
| --- | --- | --- | --- | --- | --- | --- | --- | --- |
|  | (ng/mL) | (ng/mL) | (ng/mL) | (ng/mL) | (d^−1^) | (%) |  | (ng/mL) |
| Bentazon | 0.10 | 0 | 0.085 | 0 | 0.027 | 85 | 16 | 0.008 |
| Carbamazepine | 0.10 | 0.009 | 0.106 | 0.074 | 0.56 | 97 | 16 | 0.007 |
| Diclofenac | 0.20 | 0 | 0.178 | 0 | 1.1 | 89 | 10 | 0.029 |
| Flufenacet | 0.10 | 0 | 0.026 | 0 | 0.076 | 26 | 15 | 0.016 |
| Imidacloprid | 0.10 | 0 | 0.092 | 0 | 0.14 | 92 | 16 | 0.010 |
| Metazachlor | 0.10 | 0 | 0.102 | 0.070 | 0.51 | 102 | 16 | 0.006 |
| Nicosulfuron | 0.10 | 0 | 0.107 | 0.107 | 0 | 107 | 16 | 0.017 |
| Pendimethalin | 0.10 | no data | - | - | - | - | - | - |
| Propiconazole | 0.10 | 0 | 0.032 | 0.032 | 0 | 32 | 7 | 0.014 |
| Terbuthylazine | 0.10 | 0.015 | 0.065 | 0.065 | 0 | 57 | 16 | 0.008 |
| Thiacloprid | 0.10 | 0 | 0.087 | 0.057 | 0.043 | 87 | 16 | 0.005 |
| UV 326 | 0.20 | no data | - | - | - | - | - | - |


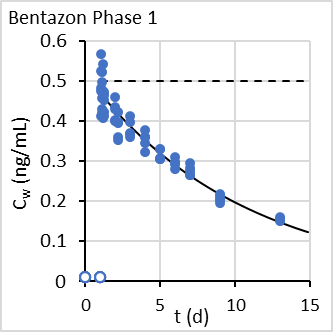

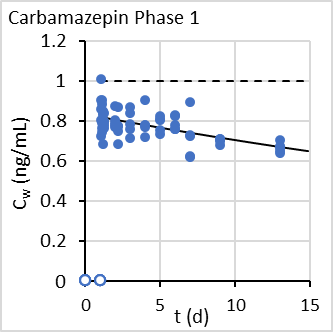

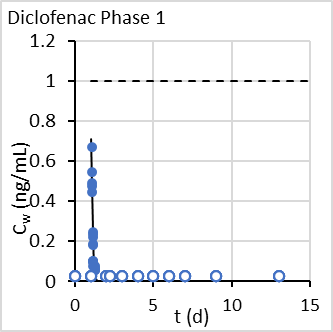


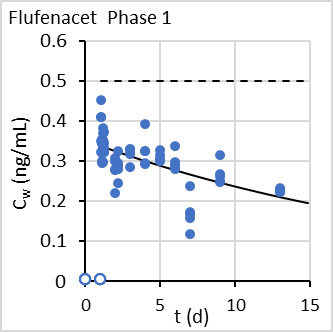

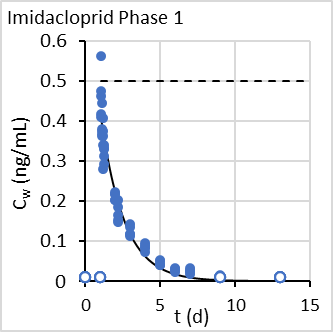

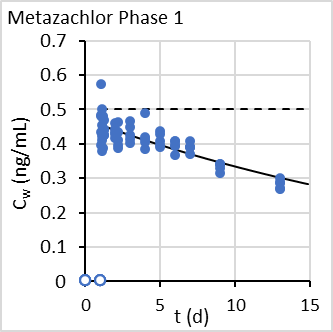


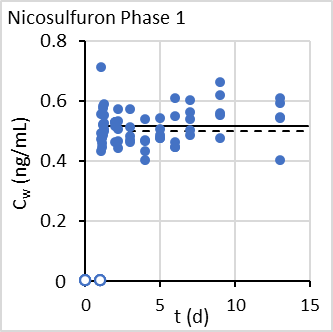

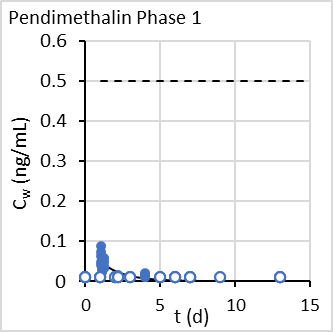

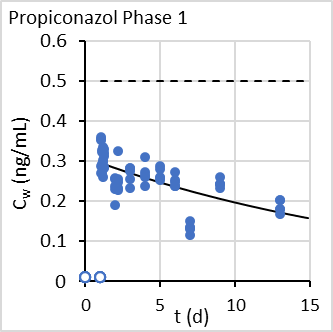


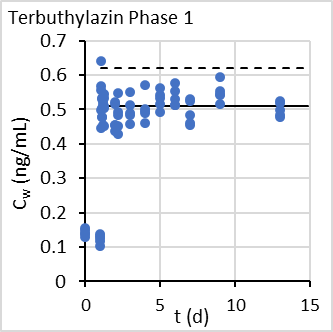

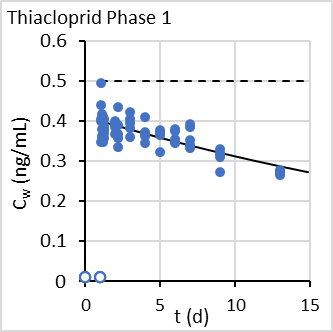

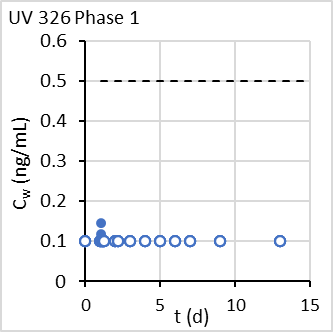


Figure S6‑1. Time evolution of aqueous concentrations (*C*_w_) in Channel A during Phase 1. Dashed lines: calculated *C*_w_ immediately after spiking (*C*_w,pre-spike_ + spike amount divided by channel volume). Solid lines: *C*_w_ model fit. Open circles: *C*_w_ values < LOQ.


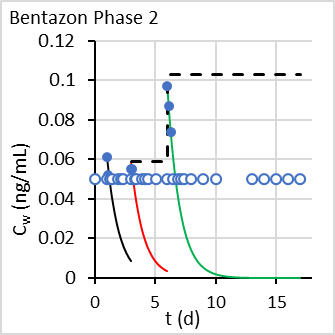

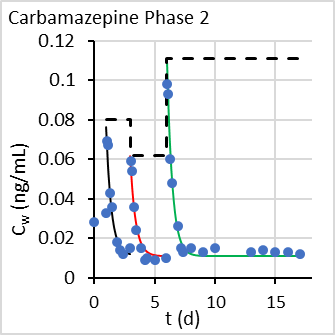

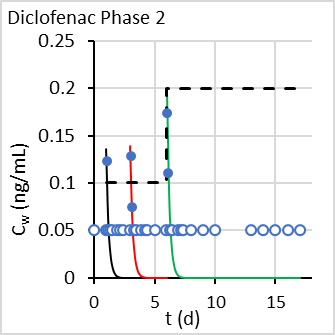


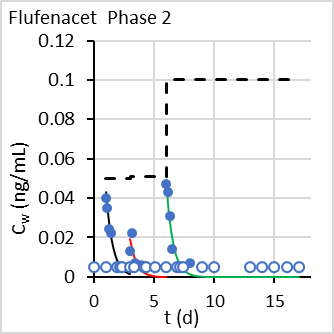

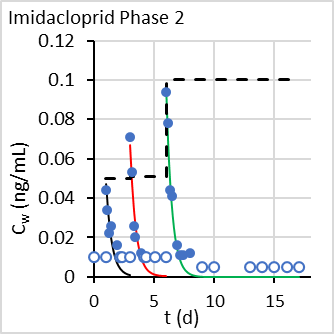

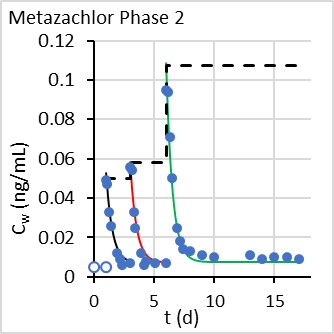


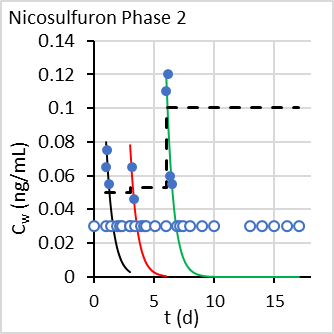

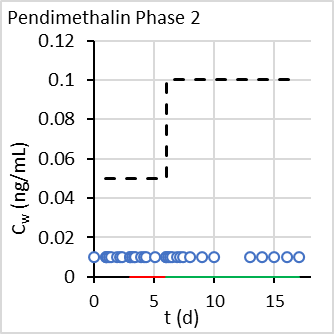

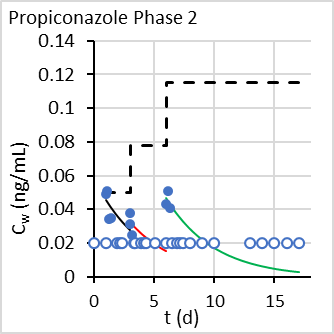


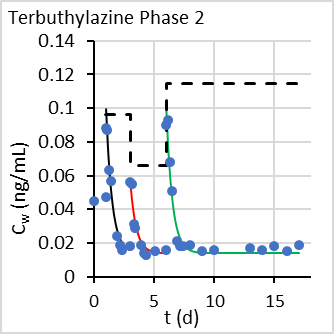

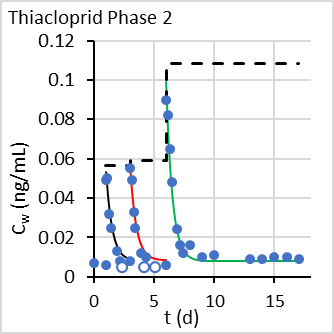

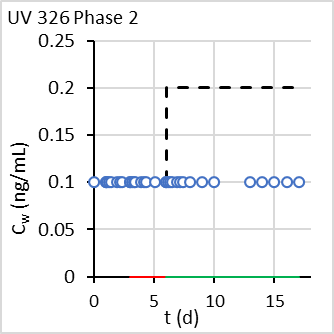


Figure S6‑2. Time evolution of aqueous concentrations (*C*_w_) in Channel A during Phase 2. Dashed lines: calculated *C*_w_ immediately after spiking (*C*_w,pre-spike_ + spike amount divided by channel volume). Solid lines: *C*_w_ model fit for spike events 1 (black), 2 (red), and 3 (green). Open circles: *C*_w_ values < LOQ.


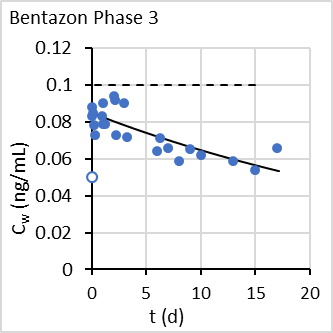

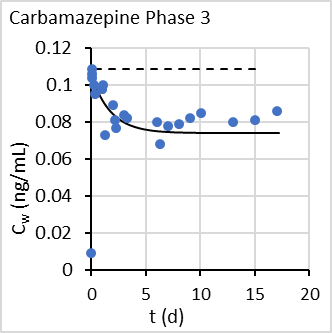

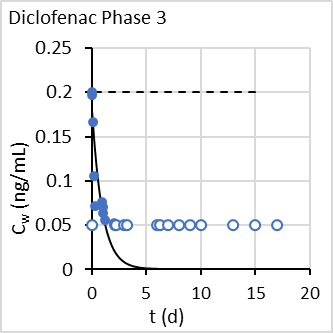


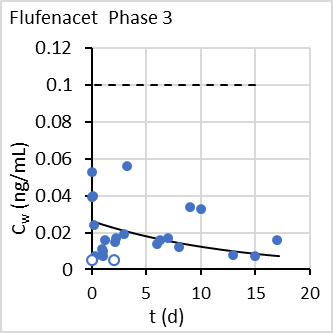

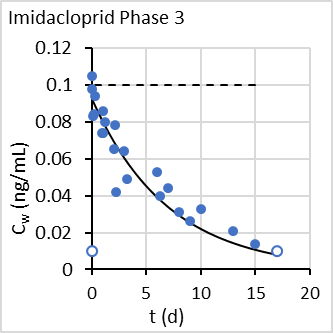

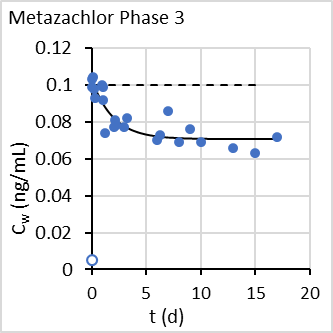


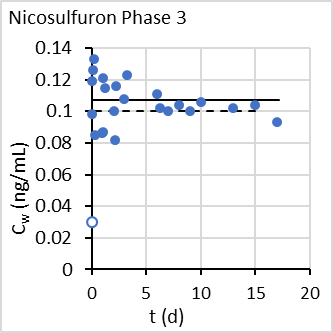

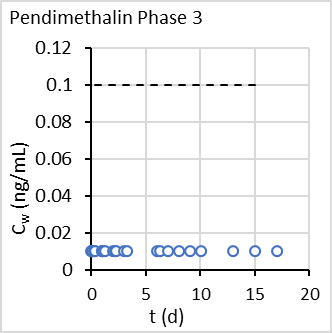

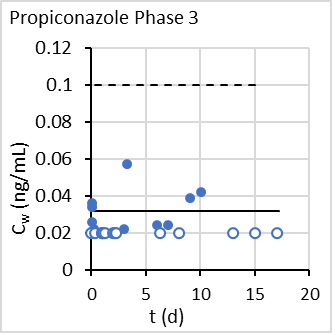


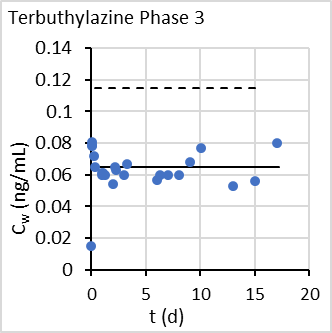

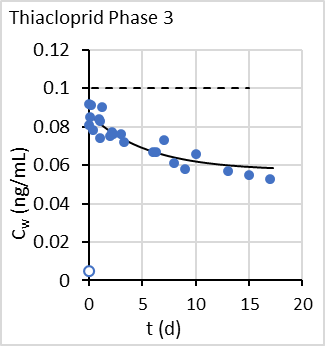

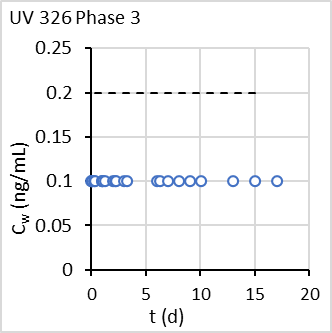


Figure S6‑3. Time evolution of aqueous concentrations (*C*_w_) in Channel A during Phase 3. Dashed lines: calculated *C*_w_ immediately after spiking (*C*_w,pre-spike_ + spike amount divided by channel volume). Solid lines: *C*_w_ model fit. Open circles: *C*_w_ values < LOQ.

# Model plots for sampling with silicone

| Diffusion model Phase 1 | *R*_s_ model Phase 1 | Diffusion model Phase 2 | *R*_s_ model Phase 2 |
| --- | --- | --- | --- |
| 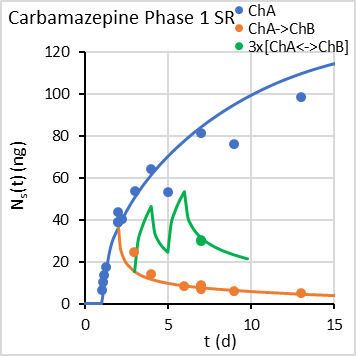  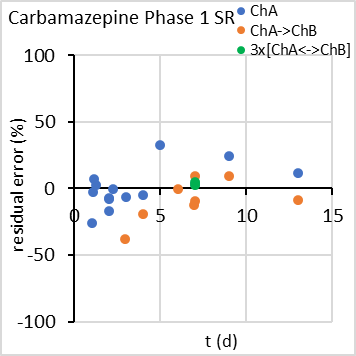 | 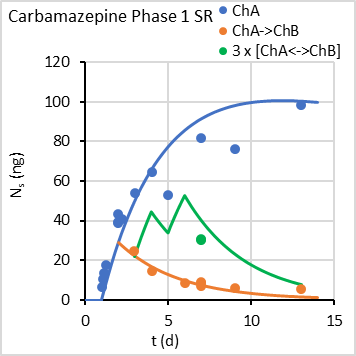  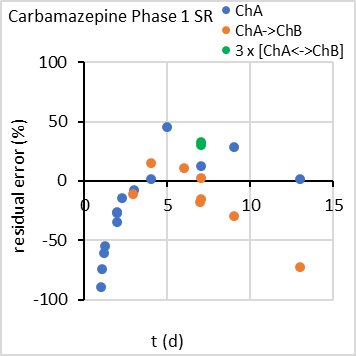 | 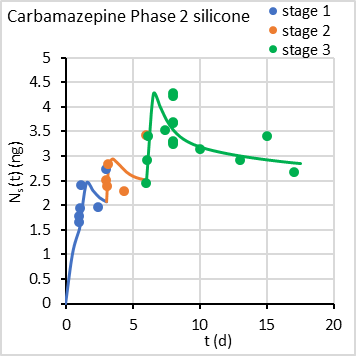  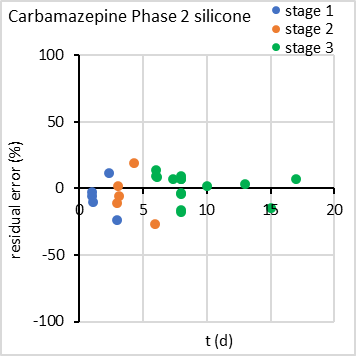 | 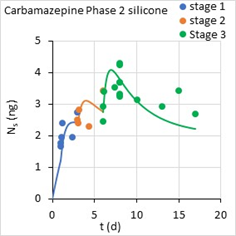  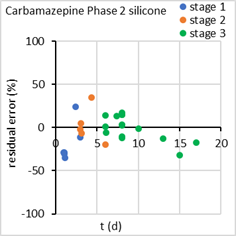 |
| 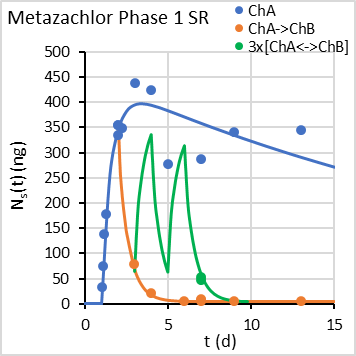  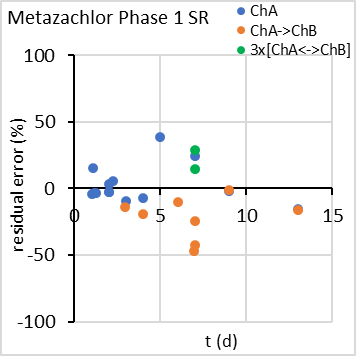 | 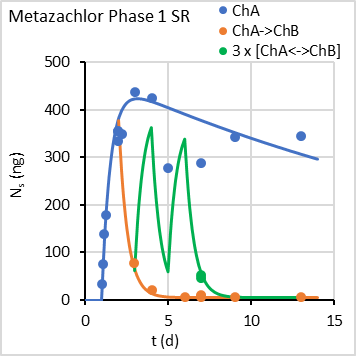  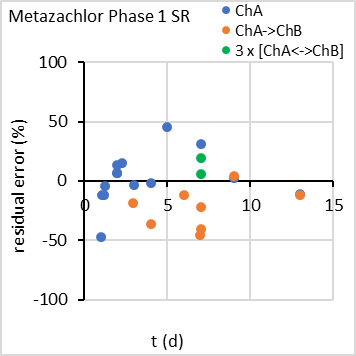 | 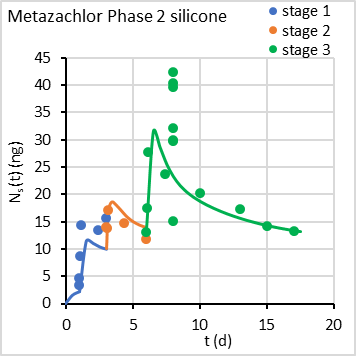  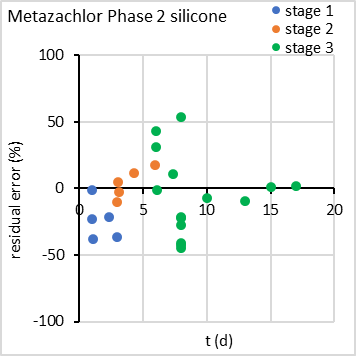 | 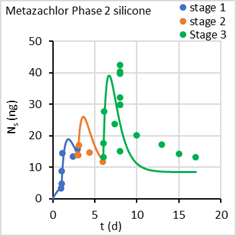  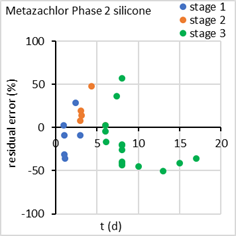 |
| 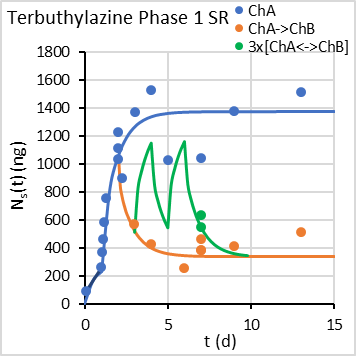  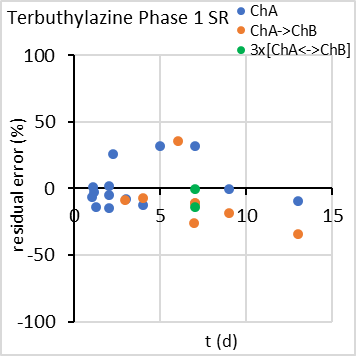 | 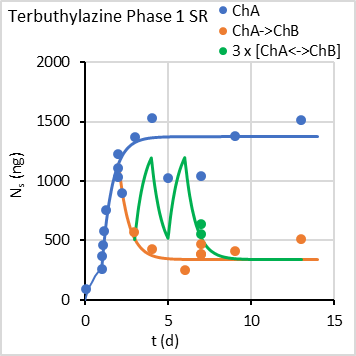  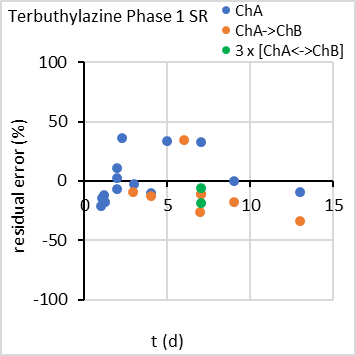 | 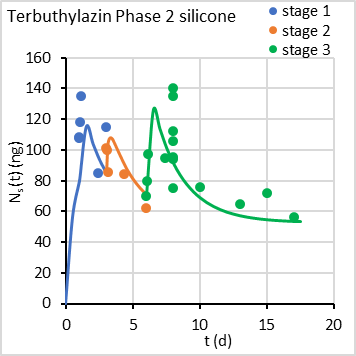  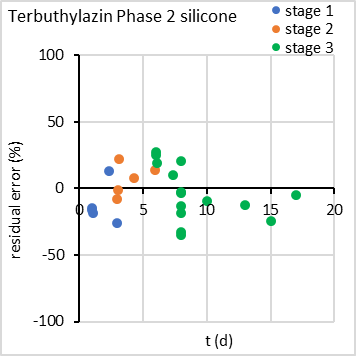 | 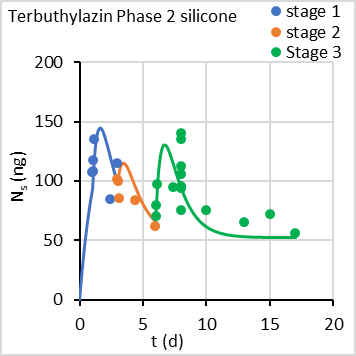  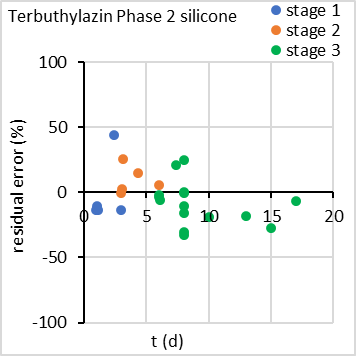 |

Figure S7‑1. Model plots and residual errors for diffusion model and *R*_s_ model for accumulation by silicone samplers during experimental phases 1 and 2. (continued...)

| Diffusion model Phase 1 | *R*_s_ model Phase 1 | Diffusion model Phase 2 | *R*_s_ model Phase 2 |
| --- | --- | --- | --- |
| 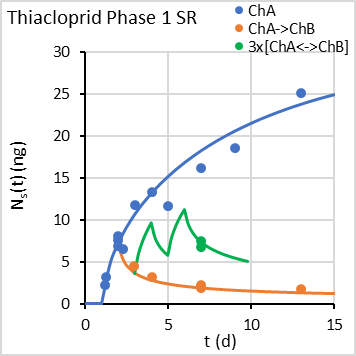  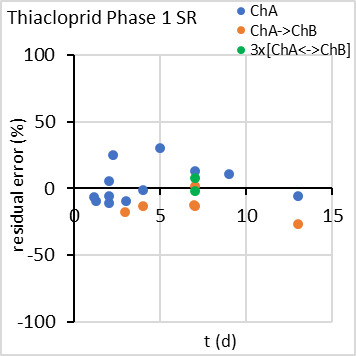 | 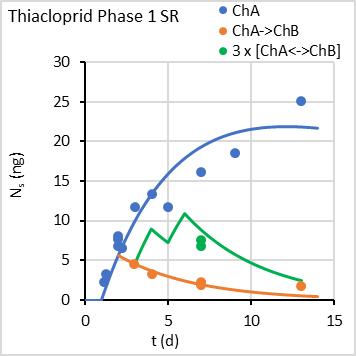  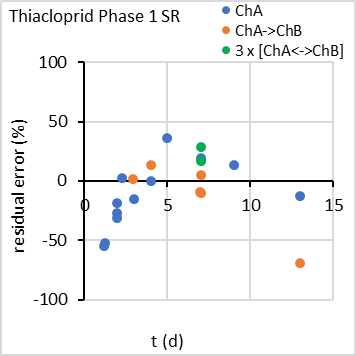 | 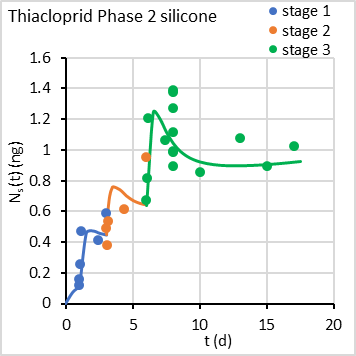  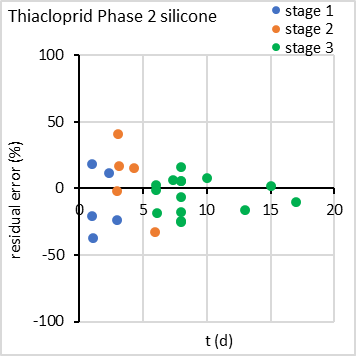 | 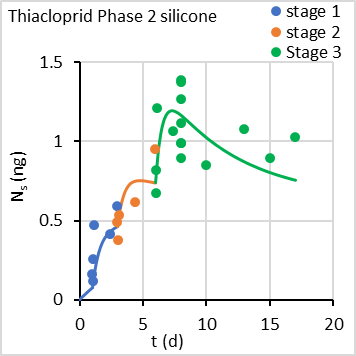  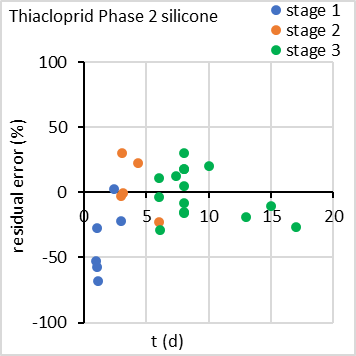 |

Figure S7‑1 (continued) Model plots and residual errors for diffusion model and *R*_s_ model for accumulation by silicone samplers during experimental phases 1 and 2.

# Flow effects for silicone samplers


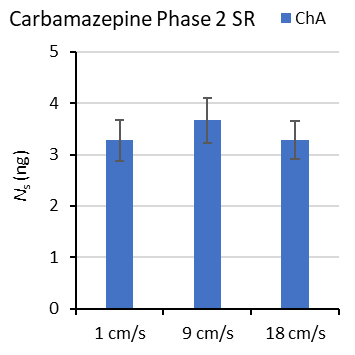

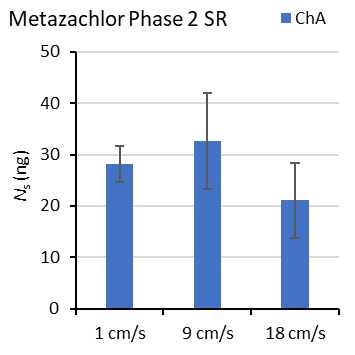


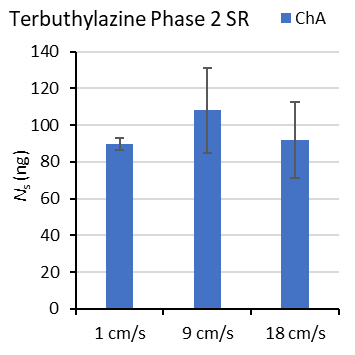

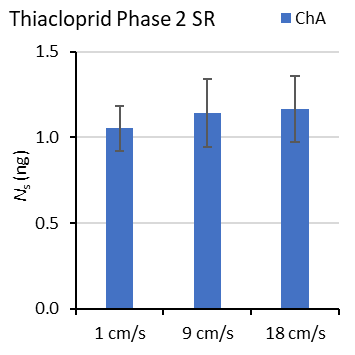


Figure S8‑1. Accumulation of target compounds by silicone samplers at water flow velocities of 1, 9, and 18 cm/s during experimental phase 2. Error bars represent standard deviations.

# Model plots for sampling by SDB-RPS samplers without membrane

| Diffusion model Phase 1 | *R*_s_ model Phase 1 | Diffusion model Phase 2 | *R*_s_ model Phase 2 | Diffusion model Phase 2 | *R*_s_ model Phase 3 |
| --- | --- | --- | --- | --- | --- |
| 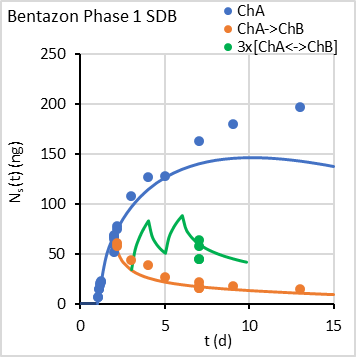  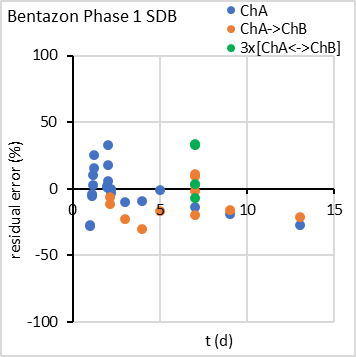 | 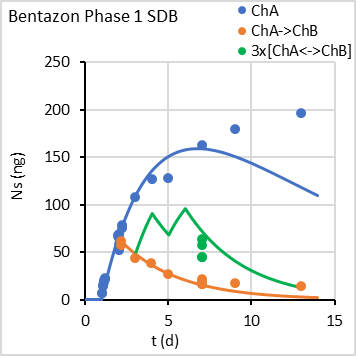  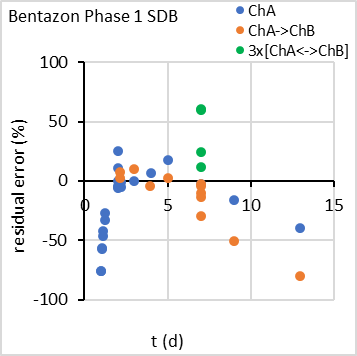 | 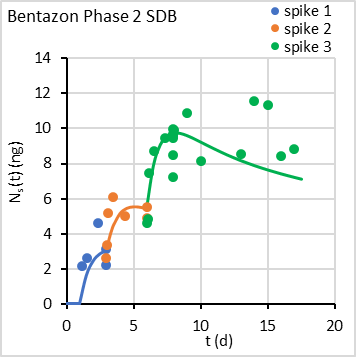  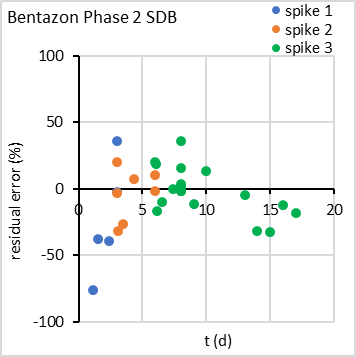 | 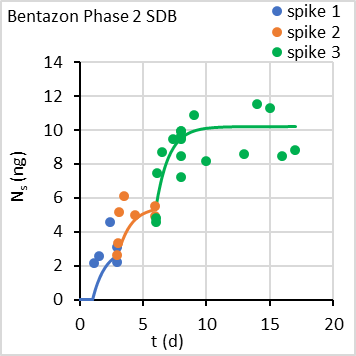  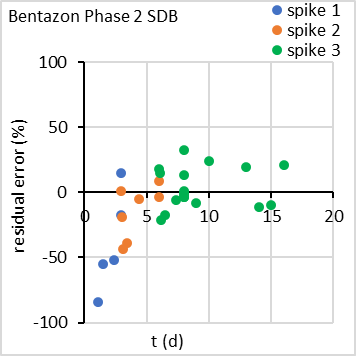 | 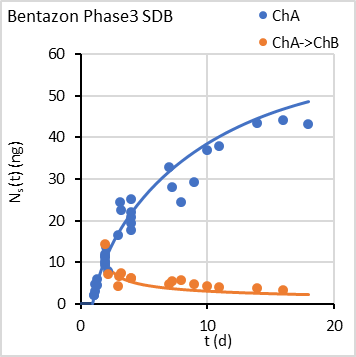  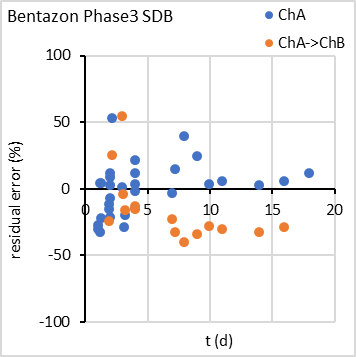 | 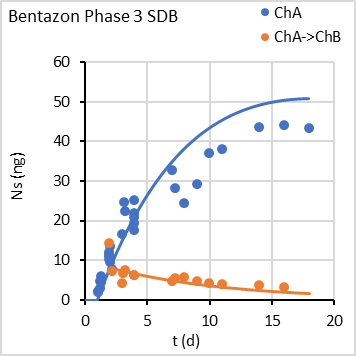  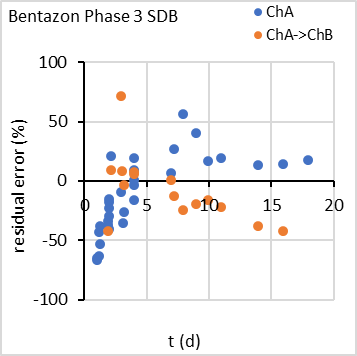 |
| 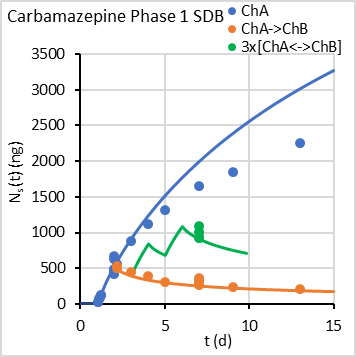  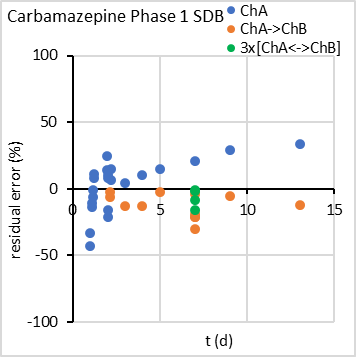 | 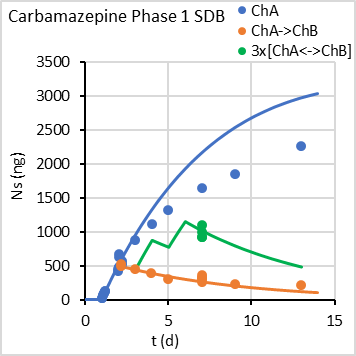  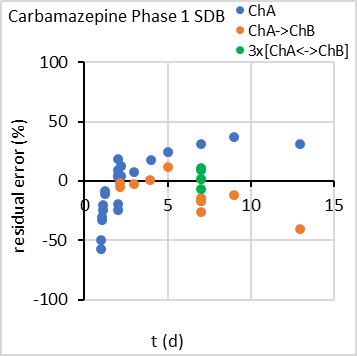 | 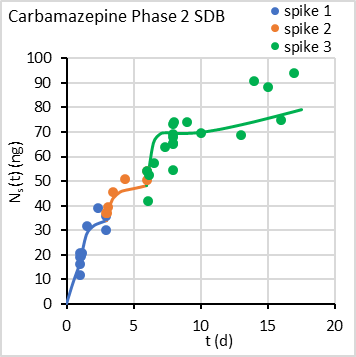  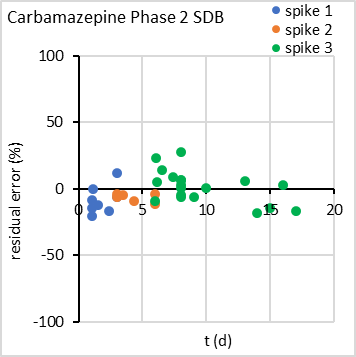 |  |  |  |

Figure S9‑1. Model plots and residual errors for diffusion model and *R*_s_ model for accumulation by SDB-RPS samplers during experimental phases 1, 2, and 3. (continued...)

| Diffusion model Phase 1 | *R*_s_ model Phase 1 | Diffusion model Phase 2 | *R*_s_ model Phase 2 | Diffusion model Phase 2 | *R*_s_ model Phase 3 |
| --- | --- | --- | --- | --- | --- |
|  |  |  |  |  |  |
|  |  |  |  |  |  |

Figure S9‑1 (continued) Model plots and residual errors for diffusion model and *R*_s_ model for accumulation by SDB-RPS samplers during experimental phases 1, 2, and 3.

(continued...)

| Diffusion model Phase 1 | *R*_s_ model Phase 1 | Diffusion model Phase 2 | *R*_s_ model Phase 2 | Diffusion model Phase 3 | *R*_s_ model Phase 3 |
| --- | --- | --- | --- | --- | --- |
|  |  |  |  |  |  |
|  |  |  |  |  |  |

Figure S9‑1 (continued) Model plots and residual errors for diffusion model and *R*_s_ model for accumulation by SDB-RPS samplers during experimental phases 1, 2, and 3.

(continued...)

| Diffusion model Phase 1 | *R*_s_ model Phase 1 | Diffusion model Phase 2 | *R*_s_ model Phase 2 | Diffusion model Phase 3 | *R*_s_ model Phase 3 |
| --- | --- | --- | --- | --- | --- |
|  |  |  |  |  |  |

Figure S9‑1. (continued) Model plots and residual errors for diffusion model and *R*_s_ model for accumulation by SDB-RPS samplers during experimental phases 1, 2, and 3.

# Flow effects for SBD-RPS samplers

Figure S10‑1. Amounts in SDB-RPS samplers at flow velocities of 1, 9, and 18 cm/s during experimental phases 1 and 2. Error bars represent standard deviations.

Table S10‑1. Slopes of log(Amount) versus log(Flow velocity) for SDB-RPS samplers.

|  | Phase 1 | Phase 1 | Phase 2 |
| --- | --- | --- | --- |
|  | ChA | ChA->ChB | ChA |
| Bentazon | 0.21 | 0.03 | 0.12 |
| Carbamazepine | 0.46 | 0.26 | 0.31 |
| Imidacloprid | 0.41 | 0.19 | 0.20 |
| Metazachlor | 0.53 | 0.33 | 0.34 |
| Nicosulfuron | 0.30 | 0.00 | 0.22 |
| Terbuthylazine | 0.51 | 0.33 | 0.38 |
| Thiacloprid | 0.52 | 0.28 | 0.34 |

# Membrane effects for SDB-RPS samplers

Table S11‑1. Model results for SDB-RPS samplers with PES membranes, without lag times. Results for nicosulfuron are omitted because of the erratic responses for this compound (Figure S11‑1).

|  | Diffusion model | | | | |  | *R*_s_ model | | | | |
| --- | --- | --- | --- | --- | --- | --- | --- | --- | --- | --- | --- |
|  | log*K*'_sw_  (mL/mL) | SE | *k*_w+m_  (um/s) | SE | s  (%) | n | log*K*_sw_  (mL/g) | SE | *R*_s_/A  (L/[dm2 d]) | SE | s  (%) |
| Bentazon | 3.73 | 0.06 | 0.98 | 0.05 | 9 | 18 | 3.78 | 0.05 | 0.63 | 0.03 | 14 |
| Carbamazepine | ns)^1^ | ns | 0.97 | 0.03 | 13 | 19 | ns)^1^ | ns | 0.83 | 0.03 | 13 |
| Imidacloprid | ns)^1^ | ns | 0.83 | 0.03 | 16 | 16 | ns)^1^ | ns | 0.71 | 0.03 | 16 |
| Metazachlor | ns)^1^ | ns | 0.91 | 0.02 | 11 | 20 | ns)^1^ | ns | 0.77 | 0.02 | 11 |
| Terbuthylazine | ns)^1^ | ns | 0.66 | 0.04 | 26 | 18 | ns)^1^ | ns | 0.57 | 0.04 | 26 |
| Thiacloprid | ns)^1^ | ns | 0.53 | 0.05 | 38 | 18 | ns)^1^ | ns | 0.45 | 0.04 | 38 |

|  |  |  |
| --- | --- | --- |

Figure S11‑1. Accumulation in SDB-RPS disks (filled circles) and PES membranes (open circles), during exposure in Channel A (blue) and Channel B (amber). Diffusion model results are shown as solid lines. (continued...)

|  |  |  |
| --- | --- | --- |
|  |  |  |

Figure S11‑1 (continued) Accumulation in SDB-RPS disks (filled circles) and PES membranes (open circles), during exposure in Channel A (blue) and Channel B (amber). Diffusion model results are shown as solid lines.

|  |  |  |
| --- | --- | --- |

Figure S11‑2. Model plots for SDB-RPS samplers with PES membranes, using the *R*_s_ model that includes lag times. Results are only shown for compounds for which a statistically significant lag time was found.

# Biofouling effects for SDB-RPS samplers

|  |  |  |
| --- | --- | --- |
|  |  |  |
|  |  |  |

Figure S12‑1. Amounts in SDB-RPS samplers after pre-fouling for 0, 2, and 4 wk, followed by exposure for 2 and 4 d.

Figure S12‑2. SDB-RPS disks after pre-fouling for 0, 2, and 4 wk.

# References

Hahn, D.W., Özişik, M.N., 2012. Heat Conduction., 3rd ed. John Wiley & Sons, Inc.

Mikhailov, M.D., Özişik, M.N., 1984. Unified analysis and solutions of heat and mass diffusion. John Wiley & Sons, New York.

Vermeirssen, E.L.M., Dietschweiler, C., Escher, B.I., van der Voet, J., Hollender, J., 2012. Transfer kinetics of polar organic compounds over polyethersulfone membranes in the passive samplers POCIS and Chemcatcher. Environ. Sci. Technol. 46, 6759–6766. https://doi.org/10.1021/es3007854
